# Calcium ions tune the zinc-sequestering properties and antimicrobial activity of human S100A12†
†Electronic supplementary information (ESI) available: Complete experimental methods, Tables S1–S14 and Fig. S1–S10. See DOI: 10.1039/c5sc03655k


**DOI:** 10.1039/c5sc03655k

**Published:** 2015-10-26

**Authors:** Lisa S. Cunden, Aleth Gaillard, Elizabeth M. Nolan

**Affiliations:** a Department of Chemistry , Massachusetts Institute of Technology , 77 Massachusetts Avenue , Cambridge , MA 02139 , USA . Email: lnolan@mit.edu ; Fax: +1-617-324-0505 ; Tel: +1-617-452-2495

## Abstract

Human S100A12 is a host-defense protein expressed and released by neutrophils that contributes to innate immunity. Apo S100A12 is a 21 kDa antiparallel homodimer that harbors two Ca(ii)-binding EF-hand domains per subunit and exhibits two His_3_Asp motifs for chelating transition metal ions at the homodimer interface. In this work, we present results from metal-binding studies and microbiology assays designed to ascertain whether Ca(ii) ions modulate the Zn(ii)-binding properties of S100A12 and further evaluate the antimicrobial properties of this protein. Our metal-depletion studies reveal that Ca(ii) ions enhance the ability of S100A12 to sequester Zn(ii) from microbial growth media. We report that human S100A12 has antifungal activity against *Candida albicans*, *C. krusei*, *C. glabrata* and *C. tropicalis*, all of which cause human disease. This antifungal activity is Ca(ii)-dependent and requires the His_3_Asp metal-binding sites. We expand upon prior studies of the antibacterial activity of S100A12 and report Ca(ii)-dependent and strain-selective behavior. S100A12 exhibits *in vitro* growth inhibitory activity against *Listeria monocytogenes*. In contrast, S100A12 has negligible effect on the growth of *Escherichia coli* K-12 and *Pseudomonas aeruginosa* PAO1. Loss of functional ZnuABC, a high-affinity Zn(ii) import system, increases the susceptibility of *E. coli* and *P. aeruginosa* to S100A12, indicating that S100A12 deprives these mutant strains of Zn(ii). To evaluate the Zn(ii)-binding sites of S100A12 in solution, we present studies using Co(ii) as a spectroscopic probe and chromophoric small-molecule chelators in Zn(ii) competition titrations. We confirm that S100A12 binds Zn(ii) with a 2 : 1 stoichiometry, and our data indicate sub-nanomolar affinity binding. Taken together, these data support a model whereby S100A12 uses Ca(ii) ions to tune its Zn(ii)-chelating properties and antimicrobial activity.

## Abstract 2

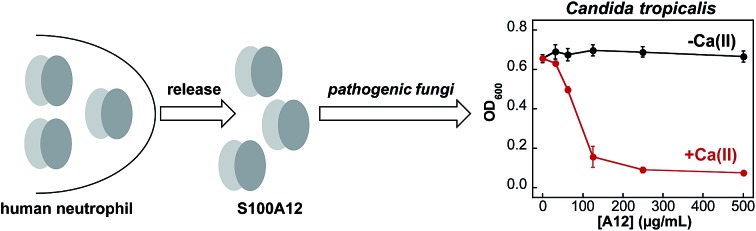
Human S100A12 exhibits Ca(ii)-dependent Zn(ii)-binding properties and antifungal activity.

## Introduction

Neutrophils are important cellular components of the innate immune system.^1^,^2^ These white blood cells are recruited to sites of infection and release antimicrobial factors into the extracellular space that include reactive oxygen and nitrogen species, proteases, antimicrobial peptides and metal-chelating proteins. In this work, motivated by the need to further elucidate the interplay between neutrophils, metal homeostasis, and microbial pathogenesis, we combine chemistry and biology to investigate a Ca(ii)- and Zn(ii)-binding protein named S100A12 that is housed in the neutrophil cytoplasm and deployed at sites of infection.

S100A12 (also termed calgranulin C, P6, CGRP, CO-Ag, CAAF-1, EN-RAGE) was first isolated from porcine granulocytes in 1994 and subsequently identified in specimens from other mammals.^3^–^9^ In 1995, the serendipitous discovery of S100A12 in human neutrophils was reported;^4^ this work revealed that S100A12 constitutes ≈5% of total cytosolic proteins in resting neutrophils, and further investigations provided its amino acid sequence and gene structure.^10^,^11^ S100A12 is associated with the inflammatory response and a variety of human pathologies that include disorders of the gastrointestinal tract (*e.g.* Crohn's disease, ulcerative colitis),^12^ type 2 diabetes,^13^ arthritis,^14^ Alzheimer's disease,^15^ and cystic fibrosis.^16^ S100A12 can interact with the receptor for advanced glycation end products (RAGE)^17^,^18^ and toll-like receptor 4 (TLR4),^19^ and the latter interaction is implicated in S100A12 activation of proinflammatory signaling in human monocytes. Moreover, S100A12 likely contributes to host defense against invading pathogens.^9^,^20^ Evidence for anti-parasitic activity was first suggested during studies of *Onchocerca volvulus* extracts obtained from tissues of onchocerciasis patients.^9^ These specimens were contaminated with various neutrophil proteins, including S100A12. Subsequent investigations demonstrated that S100A12 possesses *in vitro* anti-parasitic activity against the parasite *Brugia malayi*.^21^ Recently, elevated S100A12 levels were observed in gastric biopsy samples from patients colonized with *Helicobacter pylori*.^20^ Moreover, *in vitro* studies revealed that S100A12 has antibacterial activity against *H. pylori* and inhibits the activity of its Cag type-IV secretion system, both of which involve Zn(ii) chelation by S100A12.^20^ Taken together, these clinical and biological investigations indicate that S100A12 is an abundant and important player in human biology, and provide multiple avenues for further exploration at the molecular and physiological levels. From the perspective of metals in biology, understanding how S100A12 contributes to metal homeostasis in broad terms, and elucidating whether its metal-bound forms are relevant players in human health and disease, is of particular interest.

**Fig. 1:**
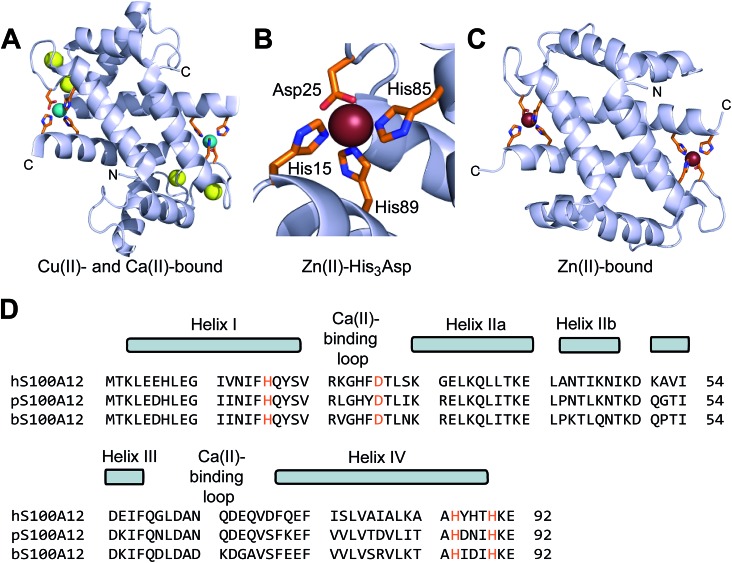
Crystal structures of metal-bound human S100A12 and amino acid sequence alignment of select S100A12 orthologues. (A) Structure of the Ca(ii)- and Cu(ii)-bound S100A12 homodimer (PDB ; 1ODB).^24^ Cu(ii) ions are shown as teal spheres, and Ca(ii) ions as yellow spheres. (B) View of the His_3_Asp motif of S100A12 taken from the structure of a Zn(ii)-bound form (PDB ; 2WCB).^25^ The Zn(ii) ion is shown as a brown sphere. (C) Structure of the Zn(ii)-bound S100A12 homodimer (PDB ; 2WCB).^25^ Zn(ii) ions are shown as brown spheres. (D) Sequence alignment of human (h), porcine (p), and bovine (b) S100A12. The secondary structural elements presented above the alignment are for the human form. The transition-metal binding residues are presented in orange.

Human S100A12 (92-residues, 10.5 kDa monomer) is a member of the S100 family of Ca(ii)-binding polypeptides that harbors two EF-hand domains and forms homooligomers. Five crystal structures of human S100A12 are available and provide insight into its oligomerization properties and coordination chemistry.^22^–^25^ The first crystal structure revealed a homodimer with four Ca(ii) ions bound at the EF-hand domains.^22^ This structure also showed two His_3_Asp motifs formed at the homodimer interface, which are comprised of His15 and Asp25 of one subunit and His85 and His89 of the other subunit. The crystal structures of Cu(ii)- and Ca(ii)-bound S100A12 and Zn(ii)-bound S100A12 established a 2 : 1 M(ii) : S100A12 homodimer stoichiometry (M = Cu, Zn) with each M(ii) ion coordinated by a His_3_Asp motif (Fig. 1).^24^ Comparison of the available apo and holo structures shows that metal binding modulates the oligomerization properties of S100A12.^22^–^25^ For instance, apo S100A12 crystallized as a dimer whereas the Zn(ii)-bound form crystallized as a tetramer.^25^ Thereafter, solution studies showed that Zn(ii) and Ca(ii) binding to S100A12 provide varying oligomeric states that include dimers, tetramers and hexamers.^26^ These studies exemplify the complex speciation of S100A12, and suggest a model whereby different forms afford functional diversity.^27^

Beyond crystallographic characterization of the Cu(ii)- and Zn(ii)-bound forms, only limited information about how S100A12 coordinates Zn(ii) and other first-row transition metal ions in solution is available, and some of the data appear contradictory. A seminal investigation of porcine S100A12 revealed stoichiometric Zn(ii) binding (*K*_d_ < 10 nM), and found that Zn(ii) coordination enhanced the affinity of this protein for Ca(ii) by several orders of magnitude.^3^ More recently, a study of Zn(ii) binding by human S100A12 reported *K*_d_ values for Zn(ii) in the micromolar range (*K*_d1_ = 16 μM, *K*_d2_ = 83 μM).^26^ Given that the pig and human forms share 70% sequence identity and have conserved His_3_Asp motifs, we had difficulty reconciling these data. Moreover, the study of human S100A12 in *H. pylori* infection supported a role for this protein in Zn(ii) sequestration,^20^ a function that requires formation of a high-affinity Zn(ii) complex.

These observations indicate that re-evaluation of the Zn(ii)-binding properties of human S100A12 is necessary. Moreover, S100A12 chelates both Ca(ii) and Zn(ii) at different sites (Fig. 1), and whether Ca(ii) ions influence the Zn(ii)-binding properties of S100A12 is unknown. In prior work, we discovered that human calprotectin (S100A8/S100A9 oligomer) uses Ca(ii) to modulate its M(ii)-binding (M = Mn, Fe, Zn) properties and exhibits enhanced M(ii) affinities in the presence of high Ca(ii) concentrations.^28^–^30^ We hypothesized that S100A12 may exhibit similar behavior given its accepted function as an extracellular Zn(ii)-chelating protein.^20^,^26^

Many outstanding questions regarding the antimicrobial activity and host-defense function of S100A12 also remain. To the best of our knowledge, no extensive studies detailing the *in vitro* antimicrobial activity of S100A12 are available in the literature. This situation contrasts the wealth of data reported for other factors produced by human neutrophils, including calprotectin and the α-defensins.^31^,^32^ The *in vitro* data available for *Staphylococcus aureus* and *H. pylori* indicate that human S100A12 has strain-selective antibacterial activity and support its contribution to the host metal-withholding response.^20^,^32^

In this work, we address these issues and examine the metal-binding properties and antimicrobial activity of human S100A12 under conditions of low and high Ca(ii). In the presence of excess Ca(ii), S100A12 readily sequesters Zn(ii) from standard microbial growth media, exhibits growth inhibitory activity against a variety of pathogenic fungi, and provides antibacterial activity against the human gastrointestinal pathogen *Listeria monocytogenes*. Our data support a model where S100A12 responds to the local Ca(ii) concentration to modulate its Zn(ii)-sequestering ability and antimicrobial activity, and effectively turn on these functions in the Ca(ii)-rich environment of the extracellular space.

## Results and discussion

### Human S100A12 depletes Zn(ii) from microbial growth medium

**Fig. 2:**
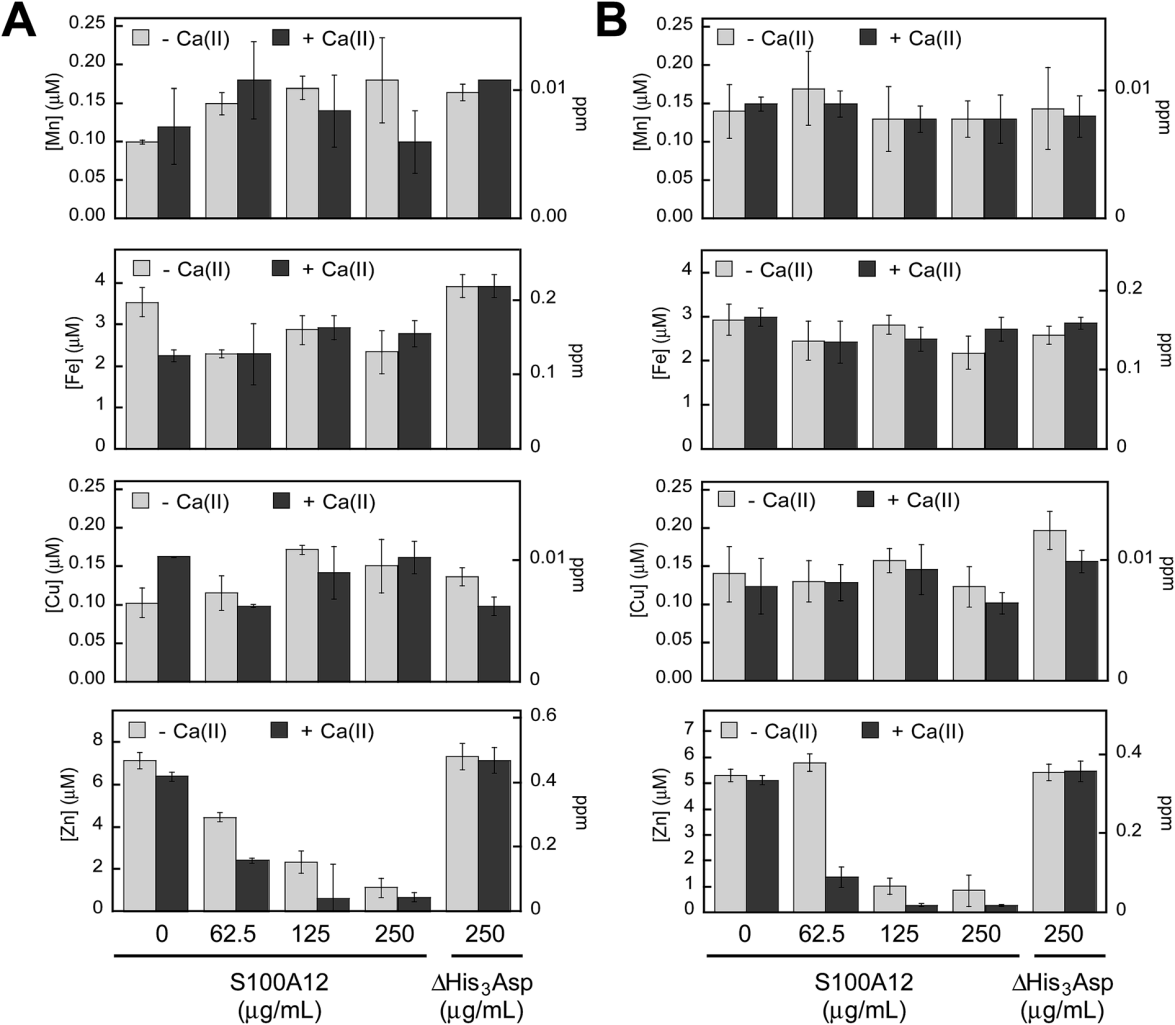
S100A12 depletes Zn(ii) from microbial growth media. (A) Metal analysis of YPD/Tris medium treated with 0–250 μg mL^–1^ S100A12 or 250 μg mL^–1^ S100A12 ΔHis_3_Asp. (B) Metal analysis of TSB/Tris medium treated with 0–250 μg mL^–1^ S100A12 or 250 μg mL^–1^ S100A12 ΔHis_3_Asp. The experiments were conducted in the absence (light gray bars) and presence (dark gray bars) of a ≈2 mM Ca(ii) supplement (mean ± SEM, *n* ≥ 3).

To probe which metal ions S100A12 sequesters from microbial growth medium, we conducted a series of metal-depletion experiments (Fig. 2 and Tables S4–S13, ESI†). We treated standard growth media employed for *in vitro* antifungal (YPD/Tris medium) or antibacterial (TSB/Tris medium) activity assays with varying concentrations of S100A12 (0–250 μg mL^–1^, *t* = 20 h, *T* = 30 °C). Following treatment of the medium with S100A12, we employed spin filtration to separate the protein from the treated medium. We quantified the amount of unbound metal in the treated medium by inductively coupled plasma-mass spectrometry (ICP-MS). Because the protein and treated medium were separated by spin filtration, this procedure does not afford an equilibrium measurement because the metal concentrations in the filtered sample are not precisely the “free” metal concentrations in a solution at equilibrium. Nevertheless, it provides an assessment of which metal ions can be sequestered by S100A12 in complex samples. To determine whether the presence of Ca(ii) influences metal sequestration, we prepared and analyzed samples with and without a ≈2 mM Ca(ii) supplement.

The YPD/Tris medium employed for antifungal activity assays (*vide infra*) contains relatively high concentrations of Fe (≈3.5 μM) and Zn (≈7 μM), and lower concentrations of Mn (≈100 nM), Co (≈130 nM), Ni (≈100 nM) and Cu (≈100 nM) (Fig. 2A and Table S4†). With the exception of Zn, S100A12 treatment had negligible effect on the concentrations of the first-row transition metals present in the YPD/Tris medium. Zinc depletion occurred, and the extent of Zn depletion depended on the concentration of S100A12 and whether the medium was supplemented with Ca(ii). The Ca(ii) effect was most obvious at 62.5 and 125 μg mL^–1^ S100A12. For instance, at 125 μg mL^–1^ S100A12, the Zn concentration in the treated medium decreased by a factor of ≈3 (–Ca) or ≈10 (+Ca).

The TSB/Tris medium employed for antibacterial activity assays (*vide infra*) also contains relatively high concentrations of Fe (≈3 μM) and Zn (≈5 μM), and the other first-row transition metals are markedly less abundant (Fig. 2B and Table S9†). S100A12 treatment resulted in Ca(ii)-dependent depletion of Zn, but not the other transition metals, from this medium. The Ca(ii) effect was most pronounced at 62.5 μg mL^–1^ S100A12 where negligible Zn(ii) depletion occurred in the absence of the Ca(ii) supplement and ≈4-fold reduction in total Zn occurred in the presence of Ca(ii).

To confirm that the His_3_Asp sites are essential for Zn(ii) depletion, we treated YPD/Tris and TSB/Tris with S100A12 ΔHis_3_Asp (250 μg mL^–1^), a variant that harbors the point mutations H15A, D25A, H85A, and H89A. This variant cannot bind metal ions at the His_3_Asp sites because the four metal-chelating residues are replaced by non-coordinating alanine moieties (ESI†). No metal depletion was observed for the ΔHis_3_Asp variant in either YPD/Tris or TSB/Tris (Fig. 2). These results demonstrate that the His_3_Asp sites are essential for Zn(ii) depletion from microbial growth media, as expected based on the Zn(ii)–S100A12 crystal structure.^25^

These metal-depletion results illuminate three key points about the metal-chelating properties of S100A12: (i) S100A12 binds Zn(ii) with sufficient affinity to deplete it from growth medium; (ii) S100A12 appears to select for Zn(ii) over other first-row transition metal ions, at least under these experimental conditions; and (iii) S100A12 uses Ca(ii) ions to modulate its Zn(ii)-binding properties, and the presence of Ca(ii) results in more effective Zn depletion from the medium. Moreover, the TSB/Tris metal-depletion profile of S100A12 is markedly different from that of calprotectin, a metal-sequestering S100 family member that harbors two different sites for transition metal ions (His_3_Asp and His_4_/His_6_) and reduces the levels of Mn, Fe, Ni, Cu, and Zn from this medium.^30^ The apparent Zn(ii) selectivity of S100A12 is consistent with prior solution studies of this protein, which provided no evidence for the formation of high-affinity Mn(ii)–S100A12 and Fe(ii)–S100A12 complexes in the absence or presence of Ca(ii).^30^,^33^ The selective Zn(ii) depletion is also reminiscent of studies of a ΔHis_4_ variant of calprotectin that only harbors an interfacial His_3_Asp site for chelating transition metals. This variant only depleted Zn(ii) from TSB/Tris mixtures.^30^

### Human S100A12 exhibits calcium-dependent antifungal activity

**Fig. 3:**
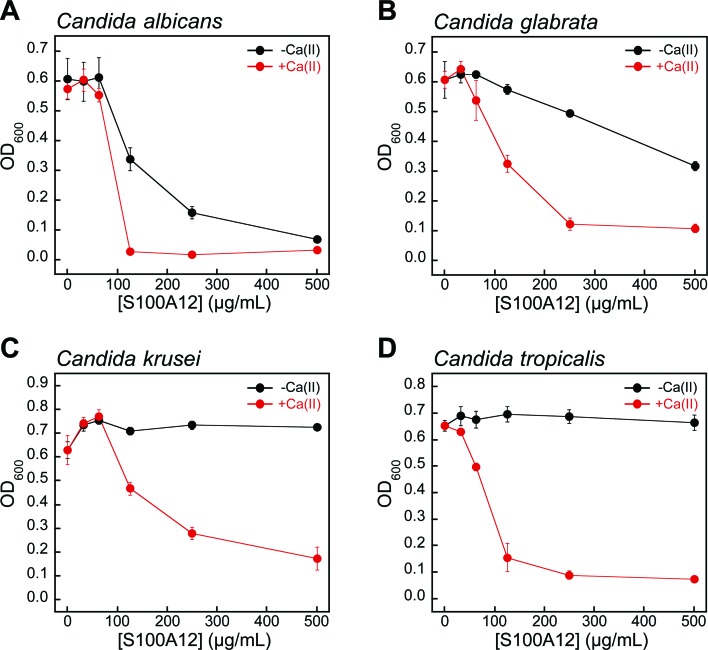
Human S100A12 exhibits Ca(ii)-dependent antifungal activity in YPD/Tris medium. (A) *C. albicans* SC 5314, (B) *C. glabrata* ATCC 200918, (C) *C. krusei* ATCC 200917, and (D) *C. tropicalis* ATCC MYA-3404. Black, *Candida* spp. treated with S100A12 in the absence of a Ca(ii) supplement. Red, *Candida* spp. treated with S100A12 in the presence of a ≈2 mM Ca(ii) supplement. The OD_600_ values were recorded at *t* = 30 h (mean ± SEM, *n* = 3).

Fungi have a significant Zn(ii) requirement, and Zn(ii) acquisition by fungal pathogens is important for virulence.^34^–^40^ We therefore questioned whether S100A12 exhibits antifungal activity that results from its ability to bind Zn(ii). To the best of our knowledge, an antifungal activity for S100A12 has not been reported. We evaluated the growth inhibitory activity of S100A12 against four *Candida* strains that cause human disease. *C. albicans* is a commensal organism of the human gastrointestinal tract and opportunistic human pathogen that can invade the mucosa and cause bloodstream infections.^41^,^42^ *C. krusei*, *C. glabrata*, and *C. tropicalis* are emerging human health threats, form biofilms, and exhibit resistance to antifungals such as the fluconazoles.^43^–^45^ When cultured in the YPD/Tris medium employed in the metal-depletion studies (Fig. 2), each fungal strain grew to an OD_600_ value of ≈0.6 in the absence and presence of a ≈2 mM Ca(ii) supplement (Fig. 3 and S5†). Addition of S100A12 (0–500 μg mL^–1^) to *C. albicans* resulted in concentration-dependent growth inhibition, and this effect was enhanced when Ca(ii) was present in the medium (Fig. 3A). The Ca(ii) enhancement was most striking at 125 μg mL^–1^ S100A12 where the OD_600_ value decreased to ≈0.3 in the absence of Ca(ii), and negligible *C. albicans* growth was observed when Ca(ii) was included in the medium.

S100A12 also displayed Ca(ii)-dependent growth inhibitory activity against *C. krusei*, *C. glabrata*, and *C. tropicalis* (Fig. 3B–D). As observed for *C. albicans*, the presence of the Ca(ii) supplement enhanced the antifungal activity of S100A12 against these three strains. Moreover, strain-to-strain variability in S100A12-mediated growth inhibition occurred in both the absence and presence of the Ca(ii) supplement. In the absence of a Ca(ii) supplement, *C. albicans* and *C. glabrata* were more susceptible to S100A12 than *C. krusei* and *C. tropicalis*. Indeed, neither *C. krusei* nor *C. tropicalis* exhibited growth inhibition in the presence of 500 μg mL^–1^ S100A12 when the Ca(ii) supplement was omitted, whereas *C. albicans* and *C. glabrata* growth was inhibited to varying degrees. Taken together, these data establish that (i) S100A12 exhibits *in vitro* antifungal activity against a number of *Candida* strains, (ii) enhanced fungal growth inhibition occurs under conditions of high Ca(ii), and (iii) the susceptibility of *Candida* spp. to S100A12 is strain-dependent.

### The His_3_Asp motifs are essential for antifungal activity

**Fig. 4:**
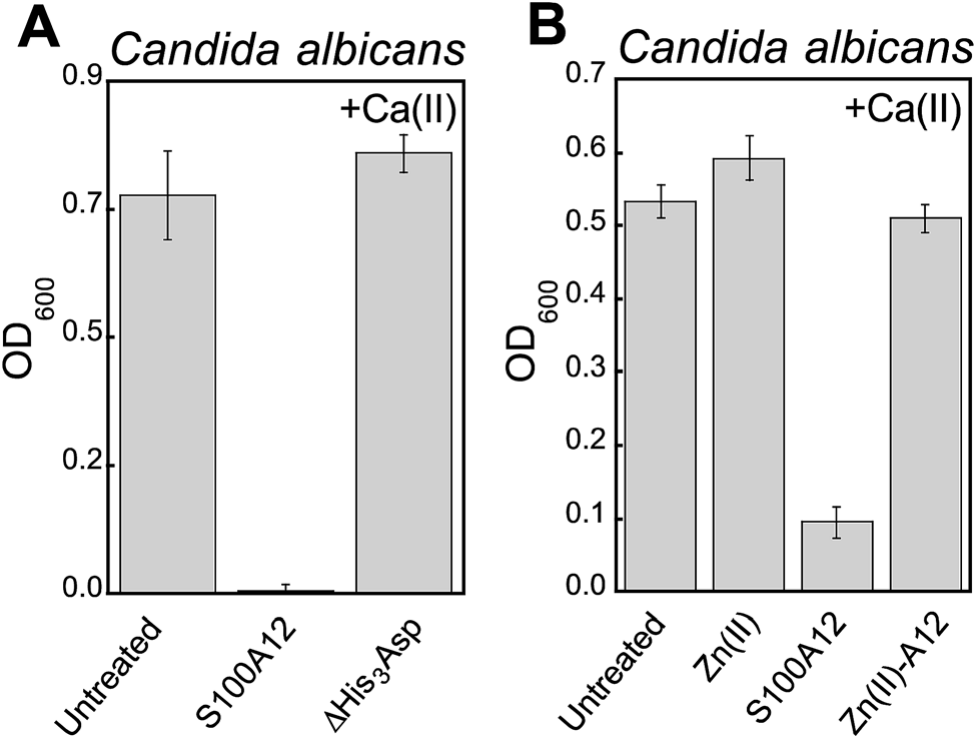
The His_3_Asp motifs of S100A12 are essential for antifungal activity against *C. albicans*. (A) Antifungal activity of 1000 μg mL^–1^ S100A12 and S10012 ΔHis_3_Asp (*t* = 20 h, mean ± SEM, *n* = 3). (B) Antifungal activity of 125 μg mL^–1^ S100A12 and Zn(ii)–S100A12 (*t* = 30 h, mean ± SEM, *n* = 3). The Zn(ii)–S100A12 sample was prepared by pre-incubating S100A12 with 2 equiv. of Zn(ii) (11.9 μM) prior to the assay. The bar labeled “Zn(ii)” indicates the growth of a culture where 11.9 μM Zn(ii) was added to the YPD/Tris medium. For each assay, the YPD/Tris medium contained a ≈2 mM Ca(ii) supplement.

We reasoned that the Ca(ii)-dependent antifungal activity of S100A12 results from Zn(ii) sequestration. We performed two additional assays to test this hypothesis and evaluate the requirement of the His_3_Asp sites for the antifungal activity against *C. albicans*. First, to determine whether the His_3_Asp sites are essential, we compared the antifungal activity of S100A12 and the ΔHis_3_Asp variant (Fig. 4A). This assay revealed that S100A12 ΔHis_3_Asp (1 mg mL^–1^, +Ca) has no effect on *C. albicans* growth relative to the untreated control. Next, to confirm that the antifungal activity originates from apo His_3_Asp sites of S100A12 and not a transition-metal-bound form, we pre-incubated S100A12 with two equivalents of Zn(ii) to afford Zn(ii)–S100A12. Similar to the ΔHis_3_Asp variant, Zn(ii)–S100A12 (125 μg mL^–1^, +Ca) did not inhibit *C. albicans* growth (Fig. 4B).

A comparison of the S100A12 concentrations (125 μg mL^–1^, ≈5 μM S100A12 or ≈10 μM His_3_Asp sites) required for maximum antifungal activity with the Zn(ii) content (≈7 μM) in the YPD/Tris medium is informative. This comparison indicates that an excess of Zn(ii)-binding sites relative to the Zn(ii) content in the growth medium is required for S100A12 to exert its full growth inhibitory activity against *Candida* spp. under these assay conditions. The metal-depletion studies indicated that 125 μg mL^–1^ S100A12 reduces the Zn level in YPD/Tris to baseline (≈620 nM) when Ca(ii) is present in the medium. Taken together, these observations suggest that the *Candida* strains evaluated in this work compete with S100A12 for Zn(ii) available in the growth medium.

### Human S100A12 exhibits strain-specific antibacterial activity

**Fig. 5:**
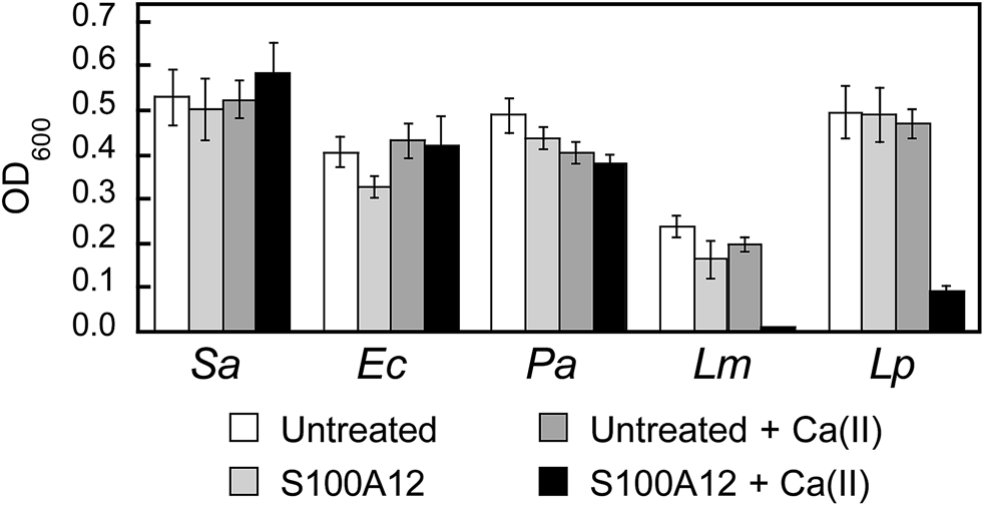
Antibacterial activity of human S100A12 (1000 μg mL^–1^) against *S. aureus* ATCC 25923 (*Sa*), *E. coli* K-12 (*Ec*), *P. aeruginosa* PAO1 (*Pa*), *L. monocytogenes* ATCC 19115 (*Lm*), and *L. plantarum* WSCF1 (*Lp*) in the absence and presence of a ≈2 mM Ca(ii) supplement. The assays were performed in TSB/Tris medium (*t* = 20 h, *T* = 30 °C).

To further evaluate the *in vitro* antibacterial activity of human S100A12, we examined its growth inhibitory activity against a panel of five bacterial strains that included *E. coli* K-12, *Pseudomonas aeruginosa* PAO1, *S. aureus* ATCC 25923, *Listeria monocytogenes* ATCC 19115, and *Lactobacillus plantarum* WCSF1. Because a prior investigation reported that S100A12 lacked antibacterial activity against *S. aureus* Newman,^32^ we screened for bacterial growth inhibition using a high concentration (1000 μg mL^–1^) of S100A12 in TSB/Tris or MRS/Tris (for *L. plantarum* only) medium with or without a ≈2 mM Ca(ii) supplement (Fig. 5). In agreement with prior work,^32^ we observed that S100A12 (±Ca) had negligible effect on *S. aureus* ATCC 25923 growth. Likewise, S100A12 afforded negligible growth inhibition for *E. coli* K-12 and *P. aeruginosa* PAO1. In contrast, cultures of *L. monocytogenes* and *L. plantarum* treated with S100A12 exhibited Ca(ii)-dependent growth inhibition. Negligible growth of *L. monocytogenes* occurred, and the growth of *L. plantarum* was markedly attenuated, when the medium contained the Ca(ii) supplement. On the basis of the metal-depletion studies, we reason that the strains susceptible to S100A12 are more sensitive to Zn(ii) deprivation. Future studies are required to address this notion on a strain-by-strain basis. We note that the inhibited growth of *L. plantarum* by S100A12 is in general agreement with our prior work, which found that the His_3_Asp site of calprotectin accounts for the full antibacterial activity against this strain *in vitro*.^30^ Moreover, the negligible activity of S100A12 against *S. aureus* and *E. coli* is reminiscent of the calprotectin ΔHis_4_ variant, which only has a His_3_Asp site at the S100A8/S100A9 interface and exhibits attenuated antibacterial activity against *S. aureus* and *E. coli* in mixed TSB/Tris medium.^28^,^32^

### Bacteria with defective zinc acquisition machinery are susceptible to S100A12

**Fig. 6:**
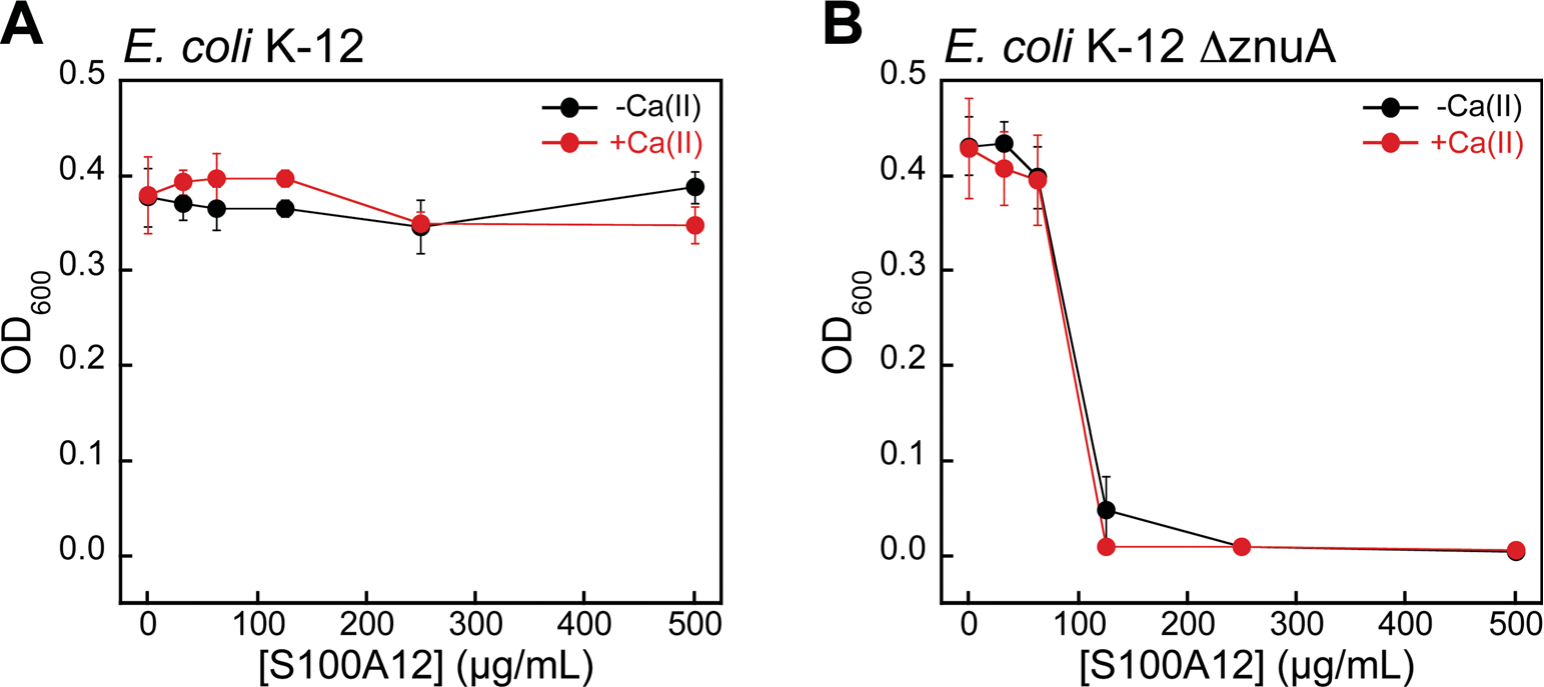
S100A12 exhibits antibacterial activity against *E. coli* with defective Zn(ii) uptake machinery in TSB/Tris medium. (A) Antibacterial activity of S100A12 against *E. coli* K-12. (B) Antibacterial activity of S100A12 against *E. coli* K-12 Δ*znuA*. Black, *E. coli* treated with S100A12 in the absence of a Ca(ii) supplement. Red, *E. coli* treated with S100A12 in the presence of ≈2 mM Ca(ii). The OD_600_ values were recorded at *t* = 20 h (mean ± SEM, *n* = 3).

To evaluate whether bacterial strains with defective Zn(ii) uptake machinery exhibit enhanced susceptibility to S100A12, we performed antibacterial activity assays with Δ*znuA* mutants of *E. coli* K-12 and *P. aeruginosa* PAO1 obtained from the Keio Collection^46^ and the Seattle *Pseudomonas aeruginosa* PAO1 Transposon Mutant Library,^47^ respectively. ZnuABC is a high-affinity Zn(ii) uptake system employed by Gram-negative bacteria.^48^–^51^ ZnuA is the periplasmic Zn(ii)-binding protein that captures Zn(ii) and delivers the ion to ZnuBC, which are located in the inner membrane.^52^ Bacterial strains with defective ZnuABC transport machinery are more susceptible to Zn(ii) deprivation than their native counterparts.^49^,^53^ Indeed, S100A12 completely attenuated the growth of *E. coli* Δ*znuA* at 125 μg mL^–1^ in both the absence and presence of a Ca(ii) supplement (Fig. 6). *P. aeruginosa* Δ*znuA* also exhibited enhanced sensitivity to S100A12; however, the growth inhibition was only partial and required the presence of Ca(ii) in the medium (Fig. S6†). A similar observation was reported in studies examining the *in vitro* antimicrobial activity of calprotectin against *P. aeruginosa* PA14.^51^ In this prior work, calprotectin did not provide complete growth inhibition of the wild-type strain or Δ*znuA* mutant; however, the growth of the Δ*znuA* strain was impaired relative to the wild-type strain.^51^ These results afforded a new hypothesis where *P. aeruginosa* PA14 has additional and as-yet undiscovered Zn(ii) acquisition machinery that allows for Zn(ii) uptake in the absence of ZnuA.^51^ Subsequent investigations of *P. aeruginosa* PAO1 and a Δ*znuA* strain cultured under conditions of low Zn(ii) revealed the expression of genes associated with additional import pathways associated with Zn(ii) acquisition as well as up-regulation of genes that encode Zn(ii)-independent proteins, which prokaryotes can employ in place of Zn(ii)-containing proteins when Zn(ii) is limited (*e.g.*, Zn(ii)-independent ribosomal proteins).^54^ The current results with S100A12 are also consistent with these studies.

### Human S100A12 is a high-affinity zinc chelator

The metal-depletion and antimicrobial activity assays presented in this work indicate that S100A12 coordinates Zn(ii) with sufficient affinity to sequester the ion and that Ca(ii) ions accentuate this function. Prior studies have examined the Zn(ii)-binding properties of S100A12 in solution. In seminal characterization of porcine S100A12, metal-binding titrations that monitored the intrinsic fluorescence of S100A12 revealed stoichiometric Zn(ii) binding (*K*_d,Zn_ ≤ 10 nM).^3^ More recently, intrinsic fluorescence was employed to study Zn(ii) chelation by human S100A12.^26^ In this work, titration of human S100A12 with Zn(ii) resulted in a gradual change in protein emission, and this titration curve was fit to afford *K*_d,Zn_ values of 16 and 83 μM for the two His_3_Asp sites.^26^ It was difficult for us to reconcile how the Zn(ii) dissociation constants for these two orthologues could vary by several orders of magnitude, and our conclusions from the metal-depletion studies and antifungal activity assays are contrary to the prior study of the human orthologue. As a consequence, we decided to further evaluate the metal-binding properties of human S100A12 in solution using techniques we employed to characterize the Zn(ii)-binding properties of calprotectin.^28^

**Fig. 7:**
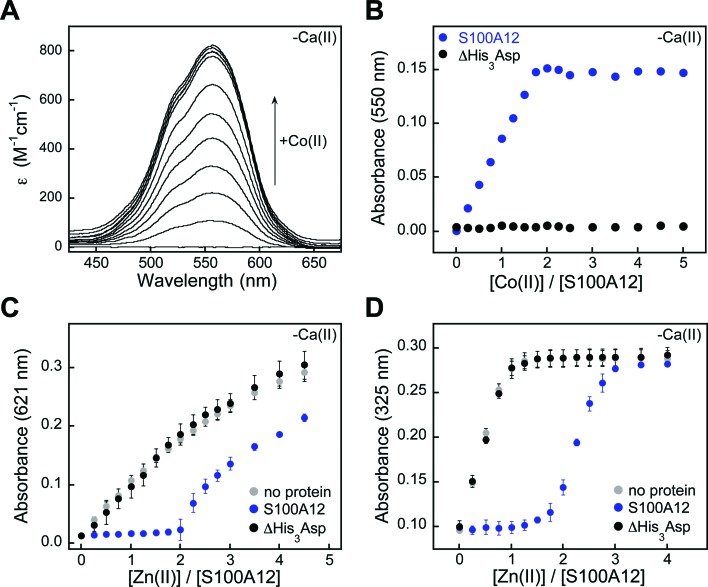
S100A12 metal-binding titrations. (A) Optical absorption spectra of S100A12 (200 μM) titrated with 0–5 equiv. of Co(ii) at pH 7.0 (75 mM HEPES, 100 mM NaCl) and 25 °C. (B) Plot of absorbance at 550 nm *versus* equivalents of Co(ii) added for titration of S100A12 or ΔHis_3_Asp (200 μM). (C) Response of Zincon (25 μM) to Zn(ii) in the presence of S100A12 or ΔHis_3_Asp (10 μM) at pH 7.0 (75 mM HEPES, 100 mM NaCl) and 25 °C (mean ± SEM, *n* = 3). The Zn(ii)–Zincon complex absorbs at 621 nm. (D) Response of MF2 (10 μM) to Zn(ii) in the presence of S100A12 or ΔHis_3_Asp (10 μM) at pH 7.0 (75 mM HEPES, 100 mM NaCl) and 25 °C (mean ± SEM, *n* = 3). The Zn(ii)–MF2 complex absorbs at 325 nm. Plots for Co(ii) titrations and Zincon competitions performed in the presence of Ca(ii) are presented in Fig. S7 and S9.†

We first utilized Co(ii) as a probe for the His_3_Asp sites because it exhibits rich spectroscopic properties as a result of its 3d^7^ electronic configuration.^55^ Addition of Co(ii) to S100A12 caused the solution to change from colorless to pink (75 mM HEPES, 100 mM NaCl, pH 7.0), and the optical absorption spectrum of Co(ii)–S100A12 exhibited ligand field transitions centered at 563 nm (*ε* = 820 M^–1^ cm^–1^) (Fig. 7A). This spectroscopic signature is reminiscent of four- or five-coordinate Co(ii) species,^55^ including Co(ii)-calprotectin where the optical features in the d–d range are dominated by Co(ii) bound at the His_3_Asp site.^28^ Moreover, Co(ii)-binding titrations revealed that the intensity of this signal increased until two equivalents of Co(ii) per S100A12 homodimer were added. The sharp inflection point indicates a 2 : 1 Co(ii)/S100A12 stoichiometry (Fig. 7B), in agreement with two His_3_Asp sites per homodimer. The Co(ii)-binding studies were performed in the absence and presence of excess Ca(ii) and comparable spectra were obtained under both sets of conditions (Fig. S7†).

Addition of Zn(ii) to Co(ii)–S100A12 resulted in loss of the absorption feature (Fig. S8†), which indicates that S100A12 binds Zn(ii) with higher affinity than Co(ii). This behavior is expected based on the Irving–Williams series.^56^ To confirm the Zn(ii)–S100A12 stoichiometry and further investigate the Zn(ii) affinity, we successfully competed S100A12 with three different chromophoric Zn(ii) chelators. Zincon is a colorimetric Zn(ii) indicator with a dissociation constant in the micromolar range (*K*_d,Zn_ ≈ 10 μM).^57^,^58^ In both the absence and presence of excess Ca(ii), no change in Zincon absorbance occurred until after two equivalents of Zn(ii) per S100A12 homodimer were added, demonstrating that S100A12 outcompeted Zincon for Zn(ii) (Fig. 7C). These titrations confirmed the expected 2 : 1 Zn(ii) : S100A12 homodimer stoichiometry and indicated that S100A12 coordinates Zn(ii) with sub-micromolar affinity. Next, we examined the competition of S100A12 with the fluorescent Zn(ii) sensors MagFura-2 (*K*_d,Zn_ ≈ 20 nM)^59^,^60^ and FluoZin-3 (*K*_d,Zn_ = 9 nM).^61^ These experiments were performed only in the absence of Ca(ii) because both MagFura-2 and FluoZin-3 exhibit a fluorescence response to this divalent cation. S100A12 outcompeted MagFura-2 (Fig. 7D) as well as FluoZin-3 (Fig. S10†), suggesting that S100A12 coordinates two equivalents of Zn(ii) with low- or sub-nanomolar affinity in the absence of Ca(ii). Taken together, the outcomes of these Zn(ii) competition titrations are in general agreement with prior characterization of porcine S100A12 where stoichiometric Zn(ii) binding (*K*_d,Zn_ < 10 nM) was observed,^3^ as well as the metal-depletion studies presented in this work.

The metal-depletion studies and antimicrobial activity assays indicate that the Zn(ii)-binding properties of S100A12 are modulated by Ca(ii) ions. In prior studies of the Zn(ii)-binding properties of human calprotectin, we employed a Ca(ii)-insensitive Zn(ii) sensor named Zinpyr-4 (ZP4, *K*_d,Zn_ = 650 pM),^62^ and discovered that calprotectin exhibits enhanced Zn(ii) affinity in the presence of Ca(ii).^28^ We sought to employ the same strategy to further elucidate how Ca(ii) modulates Zn(ii) chelation by S100A12; however, our preliminary S100A12/ZP4 experiments were plagued by artifacts that precluded using ZP4 in a quantitative or reliable manner (data not shown). In particular, mixtures of ZP4, S100A12, and Zn(ii) exhibited unstable emission. The origin of this confounding behavior is unclear, and various issues can arise when employing competitors in metal-binding titrations.^63^ We note that formation of the Zn(ii)–S100A12 complex contributed to the problem because stable emission was observed for ZP4/S100A12/Zn(ii) mixtures that contained the ΔHis_3_Asp variant. As a result, it is possible that (i) formation of ternary complexes involving Zn(ii)-bound S100A12 and ZP4 occurred, and/or (ii) Zn(ii)-dependent oligomerization of S100A12 causes ZP4 to associate with the protein. Regarding the latter scenario, the self-association properties of S100A12 are complex. Zinc binding is reported to cause the homodimer to self-associate and form tetramers and hexamers,^26^ and the Zn(ii)–S100A12 crystal structure revealed exposure of a hydrophobic patch with Zn(ii) binding.^25^ On the basis of the metal-depletion studies and antimicrobial activity assays, we expect that the presence of Ca(ii) ions increases the binding affinity of S100A12 for Zn(ii), as we discovered for calprotectin,^28^ and as-yet unidentified competitors or alternative methods are required to probe this notion further.

### Human S100A12, metal homeostasis and innate immunity

This work informs current understanding of how S100A12 contributes to metal homeostasis and innate immunity. We present the discovery that S100A12 uses Ca(ii) ions to modulate its Zn(ii)-sequestering properties and antimicrobial activity. We also report the discovery that S100A12 has *in vitro* antifungal activity. We propose this activity results from the fungi succumbing to Zn(ii) deprivation. These studies provide a guide for future investigations of human S100A12 in the contexts of the host/microbe interaction, metal homeostasis, and innate immunity.

S100A12 is co-packaged with its fellow metal-sequestering S100 protein calprotectin in the cytoplasm of human neutrophils, which indicates that the proteins are co-released at sites of infection and inflammation. Our current work illuminates similarities and differences between the metal-chelating properties of these two human S100 proteins. Both employ Ca(ii) ions to tune the affinities for transition metal ions, which are bound at sites that form at the interface between two subunits. The heterooligomeric nature of calprotectin affords two different sites at the S100A8/S100A9 interface.^64^ One site is a His_3_Asp motif similar to the sites of S100A12, whereas the other is an unusual histidine-rich motif. Like S100A12, the His_3_Asp site of calprotectin binds Zn(ii) with high affinity and has markedly lower affinity for Mn(ii) and Fe(ii).^28^–^30^ The histidine-rich motif was first identified as a His_4_ motif in the crystal structure of Ca(ii)-bound calprotectin and later determined to provide a hexahistidine coordination environment for Mn(ii) and Fe(ii).^30^,^32^,^33^,^65^ Whether this site coordinates Zn(ii) using the hexahistidine motif is currently unclear. Regardless, the histidine-rich site coordinates Mn(ii), Fe(ii) and Zn(ii) with high affinity and the metal selectivity of this site (Mn(ii) < Fe(ii) < Zn(ii)) is in agreement with the Irving–Williams series.^56^ We recently proposed that kinetics also contribute to metal sequestration at this site, enabling calprotectin to retain bound Mn(ii) and Fe(ii).^30^ On the basis of the current studies, we now question whether Zn(ii) chelation by S100A12 reduces the Zn(ii) available for capture by calprotectin in the extracellular milieu and thereby boosts the ability of calprotectin to capture other nutrient metals like Mn(ii) and Fe(ii) at the His_6_ site.

S100A12 is often described as a Cu(ii)- and Zn(ii)-chelating protein, as evidenced by crystallographic characterization (Fig. 1). The YPD/Tris and TSB/Tris growth medium employed in the metal-depletion studies contains markedly greater levels of Zn relative to Cu (Fig. 2), and we observed negligible depletion of Cu for either medium as a result of S100A12 treatment. This latter result may stem from the relative metal concentrations in the growth medium, and additional investigations are required to clarify the origin of this observation. In particular, investigations of Cu(ii)–S100A12 are limited and the Cu(ii) affinity of S100A12 is unknown, making further solution studies of copper binding to S100A12 an important avenue for future work. We note that a proposal in which Cu–S100A12 generates reactive oxygen species and thereby affords anti-parasitic activity has been put forth.^24^,^27^ To the best of our knowledge, the reported experimental support for this proposal is insufficient and limited to the fact that S100A12 can bind Cu(ii).

From the perspective of human S100A12 structure and function, the results presented in this work augment prior studies that evaluated the biophysical properties of this protein. The reported solution studies and crystallographic characterization of apo and metal-bound S100A12 delineate that the protein has the capacity to exist in many different states.^22^–^27^ This complex speciation may give S100A12 functional diversity that is important for cell signaling and the immune response.^18^,^26^,^66^ The data presented in this work that reveal S100A12 uses Ca(ii) binding to its EF-hand domains to tune its Zn(ii)-chelating properties at the His_3_Asp sites and add another dimension to this behavior. This observation is reminiscent of studies of porcine S100A12, which showed that the presence of Zn(ii) increased the Ca(ii) affinity by several orders of magnitude.^3^

Conditions of high Ca(ii) enhance the Zn(ii)-sequestering ability and antimicrobial activities of S100A12. These observations support a model whereby S100A12 responds to local Ca(ii) concentrations to tune its Zn(ii)-binding properties and are consistent with its function as an extracellular Zn(ii)-chelating protein. The extracellular space contains Ca(ii) ions at concentrations that are orders of magnitude greater than those found in the cytoplasm of resting cells.^67^ We reason that the low Ca(ii) levels in resting neutrophils allow S100A12 to maintain an apo state in the cytoplasm, and the high Ca(ii) levels in the extracellular space allow S100A12 to sequester Zn(ii) once it is deployed at a site of infection or inflammation. In prior work, we put forth the same model for the behavior of calprotectin. Whether this model is general for all S100 proteins that chelate transition metals at interfacial sites is a topic for future investigation.

## Experimental section

Complete experimental methods are provided as ESI.†

## Supplementary Material

Supplementary information
