# Supplementary material for: Calcium ions tune the zinc-sequestering properties and antimicrobial activity of human S100A12
Source: Chem Sci. 2015 Oct 26;7(2):1338–48. doi: 10.1039/c5sc03655k (PMC4762610; doi:10.1039/c5sc03655k)
Supplement: Supplementary file 1 [file SC-007-C5SC03655K-s001.pdf]

Electronic Supplementary Material (ESI) for Chemical Science.
This journal is © The Royal Society of Chemistry 2015

Supplementary Information for

Calcium Ions Tune the Zinc-Sequestering Properties and Antimicrobial Activities of Human S100A12

Lisa S. Cunden, Aleth Gaillard, and Elizabeth M. Nolan*

Department of Chemistry, Massachusetts Institute of Technology, 77 Massachusetts Avenue, Cambridge, MA 02139, USA

*Corresponding author: lnolan@mit.edu

Phone: 617-452-2495

Fax: 617-324-0505

This Supplementary Information section includes:

Design of the Synthetic Gene for S100A12 ΔHis$_3$Asp…………………………………….. S3
Complete Experimental Section…………………………………………………………… S4
Materials and General Methods……………………………………………………………….. S4
Subcloning, Overexpression and Purification of S100A12 and S100A12 ΔHisAsp………… S5
Protein Characterization……………………………………………………………………... S7
Metal Analysis (ICP-MS)……………………………………………………………………….. S7
Western blot of S100A12-treated medium (YPD)…………………………………………… S8
Antifungal Activity Assays……………………………………………………………………… S8
Antibacterial Activity Assays…………………………………………………………………… S9
Antifungal Activity Assays with Zinc Preincubation…………………………………………….. S10
Optical Absorption and Fluorescence Spectroscopy…………………………………………... S11
Zinc Competition with S100A12 and Zincon…………………………………………………… S11
Zinc Competition with S100A12 and MF2……………………………………………………… S12
Zinc Competition with S100A12 and FZ3……………………………………………..……… S12
Supplementary Tables and Figures……………………………………………………... S13
Table S1. Extinction coefficients and molecular weights of S100A12 monomers............... S13
Table S2. Mass spectrometric analysis of S100A12 and S100A12 ΔHis$_3$Asp.................... S13
Table S3. Analytical SEC elution volumes and calculated molecular weights……………… S13
Tables S4-S13. ICP-MS data from metal-depletion studies……………………………..…… S14
Table S14. Strains and growth conditions employed in this work…………………………… S17
Figure S1. SDS-PAGE of purified S100A12 and S100A12 ΔHis$_3$Asp……………………… S18
Figure S2. Analytical SEC chromatograms of S100A12 and S100A12 ΔHis$_3$Asp………… S18
Figure S3. CD spectra of S100A12 and ΔHis$_3$Asp in the absence and presence of M(II)... S19
Figure S4. Western blot of S100A12-treated media (YPD)………………………………... S19
Figure S5. Growth Curves of *Candida* spp. in the presence and absence of a Ca(II) supplement……………………………………………………………………… S20
Figure S6. Antimicrobial activity of S100A12 against *Pseudomonas aeruginosa*………... S21
Figure S7. Co(II) titrations performed in the presence of Ca(II)…………………………... S22
Figure S8. Displacement of Co(II) by Zn(II) addition………………………………………… S22
Figure S9. Zincon competition titrations performed in the presence of Ca(II)……………… S23
Figure S10. FZ3 competition experiments with S100A12 and S100A12 ΔHis$_3$Asp………. S23
Supplementary References………………………………………………………………….. S24

Design of the Synthetic Genes for Human S100A12 ΔHis$_3$Asp

The synthetic gene for human S100A12(H15A)(D25A)(H85A)(H89A) was optimized for *E. coli* codon usage and ordered from DNA 2.0. A *Nde*I restriction site was placed at the 5' end. A stop codon and a *Xho*I restriction site were placed at the 3' end. This gene was received in the pJ201 vector from DNA 2.0. The gene was sub-cloned into the *Nde*I and *Xho*I sites of pET41a to afford pET41a-*S100A12(H15A)(D25A)(H85A)(H89A)* for protein expression.

Nucleotide sequence for *Nde*I-S100A12(H15A)(D25A)(H85A)(H89A)-STOP-*Xho*I

CATATGACGAAGCTGGAAGAACACTTGGAAGGTATCGTGAACATTTTTGCCCAGTACTCCG
TTCGTAAGGGCCACTTTGCGACCCTGAGCAAGGGTGAGCTGAAACAGCTGCTGACCAAAG
AACTGGCGAATACCATTAAGAACATCAAGGACAAAGCAGTGATTGATGAGATTTTCCAGGG
CCTGGACGCGAATCAAGATGAGCAAGTTGACTTCCAAGAGTTCATCAGCCTGGTCGCAATC
GCCTTGAAAGCGGCTGCGTATCATACGGCAAAAGAG**TAA**CTCGAG

The restriction sites are underlined.
The stop codon is in bold.

Amino acid sequence for S100A12(H15A(D25A)(H85A)(H89A)

M T K L E E H L E G I V N I F A Q Y S V R K G H F A T L S K G E L K Q L L T K E L
A N T I K N I K D K A V I D E I F Q G L D A N Q D E Q V D F Q E F I S L V A I A L K
A A A Y H T A K E **Stop** L E

The residues of the restriction sites are underlined.
The mutated residues are highlighted in yellow.

Experimental Section

Materials and General Methods. All solvents and chemicals were obtained from commercial suppliers and used as received. All aqueous solutions were prepared using Milli-Q water (18.2 MΩ•cm, 0.22-μm filter).

Protein concentrations were routinely quantified by using the calculated extinction coefficients for the S100A12 homodimer (ProtParam, ε_{280} = 5960 $M^{-1}cm^{-1}$). All protein concentrations and reported stoichiometries are relative to the S100A12 homodimer (α_2).

For the microbiology assays, Luria-Bertani (LB) medium, Tryptic Soy Broth (TSB) medium, and Yeast Peptone Dextrose (YPD) medium, deMan, Rogosa and Sharpe (MRS) medium, and Brain Heart Infusion (BHI) medium, and agar plates were prepared with Milli-Q water. A Beckman Coulter DU 800 spectrophotometer thermostatted at 25 ^{o}C with a Peltier temperature controller or an Agilent 8453 diode-array spectrophotometer controlled with manufacturer-supplied software and thermostatted at 25 ^{o}C by a circulating water bath were employed for the OD_{600} measurements of bacterial and fungal cultures.

For metal-binding experiments, HEPES buffer was prepared with Ultrol grade HEPES (free acid, Calbiochem) and TraceSELECT NaCl (Sigma), and TraceSELECT aqueous NaOH (Sigma) was used to adjust the pH. To reduce metal-ion contamination, Teflon-coated or plastic spatulas were used to transfer buffer reagents, and buffers were treated with Chelex 100 resin (Bio-Rad, 10 g/L) by stirring in a polypropylene beaker for at least 1 h. The Chelex resin was removed by passing the buffer through a 0.22-μm filter, and all buffers were stored in polypropylene containers. All metal-binding studies were conducted at pH 7.0 in 75 mM HEPES, 100 mM NaCl. A Tris buffer (1 mM Tris, pH 7.5) prepared from Tris base (J. T. Baker) was used for circular dichroism (CD) spectroscopy experiments. This buffer was treated with Chelex resin for 1 h (10 g/L), filtered through a 0.22-μm filter, and the pH was readjusted to 7.0 with hydrochloric acid.

Cobalt stock solutions (100 mM) were prepared from 99.999% $CoCl_2$ hydrate (Sigma) and Milli-Q water, and zinc stock solutions (100 mM) were prepared from 99.999% anhydrous

$ZnCl_2$ (Sigma) and Milli-Q water. The metal stock solutions were prepared in acid-washed volumetric glassware and transferred to sterile polypropylene tubes for long-term storage. The working solutions were prepared by diluting the stock solutions in Milli-Q water.

FluoZin-3 (FZ3) and Mag-Fura-2 (MF2) were purchased from Invitrogen. Zincon monosodium salt was purchased from Sigma-Aldrich. Stock solutions of Zincon (≈10 mM) were prepared in anhydrous DMSO (Sigma), aliquoted into 50-μL portions and stored at -20 ^{o}C. Stocks of FZ3 (≈2 mM), and MF2 (≈2 mM), were prepared in Chelexed-treated Milli-Q water, aliquoted into 50-μL portions, and stored at -20 ^{o}C. Each aliquot was thawed only once, and experiments with these reagents were performed in the dark.

Subcloning, Overexpression, and Purification of S100A12, and S100A12 ΔHis_3Asp. The pET41a-*S100A12* expression plasmid was previously described.[1] A synthetic gene containing the *E. coli* optimized nucleotide sequence for human S100A12(H15A)(D25A)(H85A)(H89A), hereafter S100A12 ΔHis_3Asp, was obtained in the pJ201 vector from DNA 2.0 (see Design of the Synthetic Gene). The plasmid was subcloned into the *Nde*I and *Xho*I sites of pET41a using T4 DNA ligase. The plasmid identity was confirmed by DNA sequencing (MIT Biopolymers).

For protein expression, the pET41a-*S100A12* and pET41a-*S100A12(H15A)(D25A)(H85A(H89A)* expression plasmids were transformed into chemically competent *E. coli* BL21(DE3) cells prepared following standard in-house protocols. Cultures from single colonies were grown to saturation in LB medium containing 50 μg/mL kanamycin (37 ^{o}C with agitation, t ≈16-20 h), and freezer stocks were prepared by diluting the overnight cultures 1:1 (v/v) in 50% glycerol/water and stored at -80 ^{o}C.

The reconstitution and purification of human S100A12 and S100A12 ΔHis_3Asp were performed following a modified published protocol.[1] For larger-scale protein preparations, the purification protocol was modified to avoid pressure limit issues with the ÄKTA Purifier FPLC system (GE Life Sciences). The complete modified protocol is detailed below.

Frozen cell pellets (≈3 g from a 2-L culture) were thawed on ice for ≈20 min and

resuspended in 10 mL/g of lysis buffer (50 mM Tris pH 8.0, 100 mM NaCl, 1 mM EDTA, 1 mM PMSF, 0.5% Triton X-100). The resulting suspension was sonicated on ice using a Branson sonicator (40% amplitude for 2.5 min, 30 sec on / 10 sec off), and the crude lysate was clarified by centrifugation (13,000 rpm, 10 min, 4 ^{o}C). The supernatant was poured into a beaker on ice. The insoluble cell pellet was resuspended in lysis buffer (30 mL), and the sonication, centrifugation, and resuspension steps were repeated twice. The supernatant from the three rounds of lysis and centrifugation were combined, and transferred to the 4-^{o}C cold room. For cell pellets from a 2-L culture, the volume of the supernatant was typically 100 mL. To remove contaminating protein in the soluble lysate, ammonium sulfate (37.5 g for 100 mL of lysate) was slowly added over the course of 20 min with constant stirring to provide a 60% ammonium sulfate solution, and precipitation of protein was observed. S100A12 is soluble in up to 80% ammonium sulfate and therefore remains in solution. The 60% ammonium sulfate mixture was allowed to stir for an additional 1 h, and the insoluble contaminating protein fraction was pelleted by centrifugation (13,000 rpm, 10 min, 4 ^{o}C). After centrifugation, a greasy layer of precipitate remained suspended and was carefully removed using a spatula. The supernatant was dialyzed (3500 MWCO, Spectropor3) against 2 x 4 L of 20 mM HEPES, pH 8.0 at 4 ^{o}C for at least 12 h each prior to further purification.

A thin layer of precipitate formed in the dialysis tubing, and the dialysate was centrifuged (13 000 rpm, 10 min, 4 ^{o}C). The supernatant was passed through a 0.45-μm syringe filter and loaded onto a 150-mL Superloop (GE Lifesciences). Chromatographic purification of S100A12 was conducted by using an ÄKTA Purifier FPLC system (GE Life Sciences) housed in a 4-^{o}C cold room. Homodimeric S100A12 was first purified by anion-exchange chromatography using a MonoQ 10/100 GL column. A gradient of 0–10% B over 8 column volumes (Eluent A, 20 mM HEPES, pH 8.0; Eluent B, 20 mM HEPES, 1 M NaCl, pH 8.0) was employed. This step allowed for separation of homodimeric S100A12 from higher oligomeric and aggregated species of S100A12. Fractions containing S100A12 (as determined by SDS-PAGE, 15% Tris-HCl gel) were pooled, concentrated to ≈10 mL, and purified by size-exclusion chromatography (SEC)

using a HiLoad 26/600 S75 pg column (GE Lifesciences). For purified protein that was used in metal-binding experiments, the running buffer of the SEC was 20 mM HEPES, 100 mM NaCl, pH 8.0. For protein that was employed in microbiology assays, the running buffer was 20 mM Tris, 100 mM NaCl, pH 7.5. Fractions containing S100A12 were pooled and dialyzed against 1 L of the appropriate buffer (20 mM HEPES, 100 mM NaCl, pH 8.0, or 20 mM Tris, 100mM NaCl, pH 7.5) containing ≈10 g Chelex resin (Bio-Rad) at 4 ^{o}C for ≈12 h. The protein was concentrated using a 10K MWCO Amicon spin concentrator (3750 rpm, 4 ^{o}C), aliquoted into sterile microcentrifuge tubes in 100-μL portions, flash frozen in liquid N_2, and stored at -80 ^{o}C. This procedure was routinely performed using cell pellets from 2-L cultures and afforded yields of ≈100-170 mg / 2-L culture.

Protein Characterization. S100A12 and S100A12 ΔHis$_3$Asp were characterized by SDS-PAGE, mass spectrometry, analytical size exclusion chromatography, and circular dichroism (CD) spectroscopy using reported procedures.[1]

Metal Analysis (ICP-MS). To remove contaminating metals from materials used in these experiments, 5-mL centrifuge tubes (Argos Technologies Inc.) were washed with 3% HNO_3 (Sigma) and allowed to air-dry, and 4-mL spin concentrators (10K MWCO, Amicon) were washed with EDTA (300 μM in water, 1x) and Milli-Q water (3x), and allowed to dry overnight.

For these studies, two types of growth media were analyzed. The YPD/Tris medium was composed of a 32:68 (v:v) ratio of YPD and Tris buffer (20 mM Tris-HCl, 100 mM NaCl, pH 7.5). The TSB/Tris medium was composed of a 32:68 (v:v) ratio of TSB and Tris buffer.

For the metal-depletion experiments, 2.5 mL of AMA medium (either YPD/Tris or TSB/Tris) were transferred to the washed 5-mL centrifuge tubes. Protein samples were buffer exchanged into AMA buffer using 0.5-mL 10 MWCO Amicon spin concentrators, and diluted to final concentrations of 62.5, 125, 250, and 500 μg/mL and in the AMA medium. Untreated AMA medium containing no protein and the S100A12-treated media samples were incubated for 20 h (30 ^{o}C, 150 rpm). The samples were filtered using the 4-mL Amicon spin concentrators. The flow through was collected and acidified to 3% HNO_3 by addition of 70% HNO_3. Samples were

analyzed at either the Microanalysis Laboratory at the University of Illinois at Urbana-Champaign (UIUC) (one set of samples for TSB/Tris incubated with S100A12) or the Center for Environmental Health Sciences (CEHS) Core Facility at MIT (all other samples). At UIUC, the concentrations of Mn, Co, Ni, Cu, and Zn were quantified by inductively coupled plasma-mass spectrometry (ICP-MS), and the metal concentrations of Mg, Ca, and Fe were quantified by inductively coupled plasma-optical emission spectroscopy (ICP-OES). For samples analyzed at MIT, the concentrations of Mg, Ca, Fe, Mn, Co, Ni, Cu, and Zn were quantified employing an Agilent 7900 ICP-MS used in Helium mode outfitted with integrated autosampler (I-AS). An internal standard of erbium or terbium (Agilent) was used to control for sample effects, and the concentrations of analyte were calibrated using standards prepared by serial dilution of the Agilent Environmental Calibration Standard mix. All experiments were performed at least three times with four different starting stocks of media. The mean and SEM values are reported.

Western Blot. To ensure that the S100A12 present during treatment of growth medium did not pass through the spin filter, Western blot analysis was performed on the retentate and flow through obtained from each sample. Sodium dodecyl sulfate-PAGE (SDS-PAGE) was performed on a 15% glycine gel. The proteins were transferred to a nitrocellulose membrane following the manufacturer's procedure (BioRad). S100A12 was blotted with a 1:1000 dilution of monoclonal mouse IgG2b to human S100A12/EN-RAGE (161205) (R&D Systems). The antibody was blotted with a 1:10,000 dilution of infrared dye-labeled goat anti-mouse IgG (LI-COR Biosciences), and the blot was visualized using a LI-COR Odyssey Scanner.

Antifungal Activity Assays. The growth inhibitory activity of S100A12 against *Candida albicans* SC5314, *C. glabrata* ATCC 200918, *C. krusei* ATCC 200917, and *C. tropicalis* ATCC MYA-3404 was investigated by following previously published protocols for culturing these organisms.[2] Fungal strain stocks were stored at -80 ^{o}C in YPD medium containing 25% glycerol. Overnight cultures were inoculated into 10 mL of YPD in a 250-mL baffled flask from single colonies on agar plates and grown overnight (37 ^{o}C, 150 rpm, t ≈20 h) on a rotating wheel. The overnight cultures were then diluted 1:50 to a final volume of 10 mL in a 250-mL baffled flask

and allowed to grow to mid-log phase at an $OD_{600} \approx 1$ (t ≈3.5 h). The cultures at mid-log phase were diluted 1:500 into YPD/Tris medium (32:68 (v:v) ratio of YPD and Tris buffer (20 mM Tris-HCl, 100 mM NaCl, pH 7.5)), and 90 μL of the dilution added to a well containing 10 μL of the 10x protein stock in 96-well plates (Corning, Inc.). For samples containing a Ca(II) supplement, the Tris buffer was supplemented with 3 mM Ca(II). Each condition was repeated in three wells to obtain an average value for each time point for each trial. To avoid evaporation, the plate was covered with a wet paper towel, wrapped with Saran wrap. The plate was then incubated in an incubator-shaker (37 °C, 150 rpm, t ≈30 h). The growth was monitored by OD_{600} at regular intervals using a Synergy HT plate reader (BioTek). To resuspend cultures that formed clumping at the bottom of the wells, the plates were shaken vigorously prior to OD_{600} measurement. Each set of experiments was performed at least three times with at least two different protein and medium stocks. The mean and SEM are reported.

Antibacterial Activity Assays. The growth inhibitory activity of S100A12 against bacterial strains was investigated by following previously published protocols.[3,4] Bacterial strain stocks were stored at -80 °C in media containing 25% glycerol. AMA medium (TSB/Tris) was prepared using sterile technique. TSB was used for *S. aureus* ATCC 25923, *E. coli* K-12, and *P. aeruginosa* PAO1, MRS was used for *L. plantarum* WCSF1, and BHI was used for *L. monocytogenes* ATCC 19115. Overnight cultures were inoculated into 5 mL of growth medium in a culture tube (TSB, MRS, or BHI) from single colonies from agar plates (TSB, MRS, or BHI) and grown overnight (37 °C, t ≈16 h) on a rotating wheel. The overnight cultures were then diluted 1:100 in a 5-mL culture tube and allowed to grow to mid-log phase at an $OD_{600} \approx 0.6$ (t ≈2 - 4.5 h).

The assays performed with *L. plantarum* and *L. monocytogenes* were modified as follows. *L. plantarum* cultures were grown at 30 °C, 150 rpm in MRS medium, and modified AMA medium composed of MRS medium and Tris buffer (20 mM Tris-HCl, 100 mM NaCl, pH 7.5) (MRS/Tris 32:68 (v:v)) was used. *L. monocytogenes* cultures were grown at 30 °C, 150 rpm in BHI medium, and modified AMA medium composed of BHI medium and Tris buffer (BHI/Tris

32:68 (v:v)) was used. The AMA medium for *S. aureus*, *E. coli*, and *P. aeruginosa* was composed of TSB medium and Tris buffer (TSB/Tris 32:68 (v:v)). The cultures at mid-log phase were diluted 1:500 into the corresponding AMA medium, and 90 μL of the dilution added to a well containing 10 μL of the 10x protein stock. For samples containing a Ca(II) supplement, the Tris buffer was supplemented with 3 mM Ca(II). Each condition was repeated in three wells to obtain an average value for each time point for each trial. The plate was covered with a wet paper towel and wrapped with Saran wrap. The plate was then allowed to incubate in an incubator-shaker (37 ^{o}C, 150 rpm, t ≈20 h). The growth was monitored by OD_{600} at regular intervals using a plate reader. Each set of experiments was performed at least three times with at least two different protein and media stocks. The mean and SEM are reported.

Antifungal Activity Assays with Zinc Preincubation. The Zn(II) preincubation assays with *C. albicans* were performed following analogous protocols for studying the effects Mn(II) and Fe(II) addition to calprotectin.[1,4] Overnight cultures were inoculated with 10mL of YPD in a 250-mL baffled flask from single colonies on agar plates, and grown overnight on an incubator-shaker (37 ^{o}C, 150 rpm, t ≈20 h). The overnight cultures were then diluted 1:50 in a 250-mL baffled flask and allowed to reach an OD_{600} ≈1.0 after ≈3.5 h, corresponding to its mid-log phase. To prevent precipitation of protein (125 μg/mL) upon addition of 2 equiv. of Zn(II), the dilution factors were adjusted in this experiment. The mid-log phase cultures were diluted 1:56 into AMA medium (YPD/Tris containing a 2 mM Ca(II) supplement), and 10 μL of the dilution added to a well containing 90 μL of 1.1x of protein prepared in the same AMA medium, preincubated with two equivalents of Zn(II). The 96-well plate was covered with a wet paper towel, wrapped with Saran wrap, and allowed to incubate in an incubator-shaker (37 ^{o}C , 150 rpm, t ≈30 h). Fungal growth was monitored by OD_{600} at regular intervals using a plate reader. To resuspend cultures that formed clumping at the bottom of the wells, the plates were shaken vigorously prior to OD_{600} measurement. Each set of experiments was performed at least three times with at least two different protein and media stocks. The mean and SEM are reported.

Optical Absorption and Fluorescence Spectroscopy. Optical absorption spectra were collected on a Beckman Coulter DU 800 spectrophotometer thermostatted at 25 °C with a Peltier temperature controller. Fluorescence spectra were collected on a Photon Technologies International QuantaMaster 40 fluorimeter outfitted with a continuous xenon source for excitation, autocalibrated QuadraScopic monochromators, a multimode PMT detector, and a circulating water bath maintained at 25 °C. This instrument was controlled by the FelixGX software package. Quartz cuvettes with 1-cm path lengths (Starna) were employed for all optical absorption measurements. Due to the large number of cuvettes needed for the experiments with FZ3, poly(methyl methacrylate) (PMMA) cuvettes (Fisher Scientific) were employed for the fluorescence experiments with this Zn(II) sensor. All optical absorption and fluorescence spectroscopic experiments were performed at least in triplicate.

Zinc Competition with S100A12 and Zincon. A 2-mL solution containing 10 μM S100A12 or S100A12 ΔHis_3Asp and 20 μM Zincon was prepared in a quartz cuvette (75 mM HEPES, 100 mM NaCl, pH 7.0). The solution was titrated with 0-4.5 equivalents of Zn(II) (1 μL of a 2 mM $ZnCl_2$ aqueous solution per addition) at room temperature. The samples were allowed to equilibrate for 2 min after each Zn(II) addition, and the optical absorption spectra were collected from 200 to 800 nm. The absorbance at 621 nm was plotted versus the [Zn(II)] / [S100A12] ratio. Zincon has reported $K_{d,Zn}$ values of 12.6 and 5.8 μM.[5,6]

Zinc Competition with S100A12 and MF2. A 2-mL solution containing 10 μM S100A12 or S100A12 ΔHis_3Asp and 10 μM MF2 was prepared in a quartz cuvette (75 mM HEPES, 100mM NaCl, pH 7.0) and titrated with 0–4 equivalents of Zn(II) (1 μL of a 2 mM $ZnCl_2$ aqueous solution per addition) at room temperature. The samples were allowed to equilibrate for 2 min after each Zn(II) addition, and the optical absorption spectra were collected from 200 to 800 nm. The absorbance increase at 325 nm and decrease at 366 nm were plotted versus the [Zn(II)] / [S100A12] ratio. Reported $K_{d,Zn}$ values of MF-2 are 20 and 36 nM.[7, 8]

Zinc Competition with S100A12 and FZ3. Solutions (1 mL) containing FZ3 (2 μM) and S100A12 (2 μM) or S100A12 ΔHis_3Asp (2 μM) were prepared in PMMA cuvettes (75 mM

HEPES, 100 mM NaCl, pH 7.0). Each solution was mixed gently and incubated for 1 h in the dark at room temperature. The emission spectrum of each solution was then recorded. One equivalent of Zn(II) was subsequently added to each sample, and the solutions were gently mixed and incubated for 2.5 h in the dark at room temperature. The emission spectrum of each solution was then recorded. The samples were excited at 494 nm, and the emission was monitored from 500–650 nm (-Zn(II) samples) or 507–650 nm (+Zn(II) samples). Emission spectra from a representative trial are presented. The apparent $K_{d,Zn}$ of FZ3 is 9 nM.[9]

Supplementary Tables and Figures

Table S1. Molecular weights and extinction coefficients for S100A12 monomers.

Protein	Molecular Weight (Da)[a]	ε_{280} (M^{-1} cm^{-1})[b]
S100A12	10575.0	2980
S100A12 ΔHis$_3$Asp	10332.8	2980

[a] Molecular weights were calculated by using the ProtParam tool available on the ExPASy server (http://web.expasy.org/protparam). These values include the N-terminal Met residue. No *m/z* corresponding to loss of the N-terminal Met residue was observed. [b] Extinction coefficients (280 nm) were calculated by using the ProtParam tool.

Table S2. Mass spectrometric analysis of S100A12 and S100A12 ΔHis$_3$Asp.

Protein	Calculated Mass (Da)	Observed Mass (Da)
S100A12	10575.0 (monomer)	10575.6 (monomer)
	21150.0 (dimer)	21150.2 (dimer)
S100A12 ΔHis$_3$Asp	10332.8 (monomer)	10332.8 (monomer)
	20665.6 (dimer)	20665.9 (dimer)

Table S3. Analytical SEC elution volumes and calculated molecular weights.[a]

	Ca(II)	Elution Volume (mL)	Calculated Molecular Weight (kDa)
S100A12	-	12.4	24.2
S100A12	+	12.2	26.4
S100A12 ΔHis$_3$Asp	-	12.1	27.6
S100A12 ΔHis$_3$Asp	+	12.3	25.8

[a] Each sample contained 30 μM protein (75 mM HEPES, 100 mM NaCl, pH 7.0). The +Ca(II) samples contained 1.2 mM (40 equiv.) Ca(II) in the sample and running buffer. The experiments were performed at 4 °C.

Table S4. Metal analysis of untreated YPD/Tris medium in the absence and presence of a 2-mM Ca(II) supplement (mean ± SEM, *n* = 4).

**Element**	**–Ca(II), ppm**	**–Ca(II), μM**	**+Ca(II), ppm**	**+Ca(II), μM**
Mg	3.3 ± 0.2	135 ± 8	3.1 ± 0.2	130 ± 8
Ca	3.7 ± 0.2	92 ± 5	70 ± 15	1800 ± 400
Mn	0.009 ± 0.001	0.100 ± 0.002	0.006 ± 0.002	0.12 ± 0.05
Fe	0.20 ± 0.02	3.5 ± 0.4	0.124 ± 0.004	2.2 ± 0.1
Co	0.009 ± 0.001	0.14 ± 0.02	0.007 ± 0.001	0.11 ± 0.01
Ni	0.005 ± 0.002	0.09 ± 0.02	0.008 ± 0.001	0.13 ± 0.03
Cu	0.007 ± 0.001	0.10 ± 0.03	0.009 ± 0.001	0.163 ± 0.001
Zn	0.47 ± 0.02	7.2 ± 0.4	0.42 ± 0.01	6.4 ± 0.2

Table S5. Metal analysis of YPD/Tris medium treated with 62.5 μg/mL of S100A12 in the presence and absence of a 2-mM Ca(II) supplement (mean ± SEM, *n* = 4).

**Element**	**–Ca(II), ppm**	**–Ca(II), μM**	**+Ca(II), ppm**	**+Ca(II), μM**
Mg	3.1 ± 0.2	130 ± 9	3.1 ± 0.3	133 ± 7
Ca	3.5 ± 0.2	88 ± 4	3.4 ± 0.2	82 ± 3
Mn	0.009 ± 0.001	0.16 ± 0.01	0.010 ± 0.000	0.17 ± 0.02
Fe	0.22 ± 0.02	3.9 ± 0.3	0.20 ± 0.02	3.8 ± 0.03
Co	0.001 ± 0.000	0.17 ± 0.000	0.01 ± 0.004	0.17 ± 0.004
Ni	0.003 ± 0.003	0.06 ± 0.06	0.0008 ± 0.003	0.14 ± 0.06
Cu	0.008 ± 0.001	0.120 ± 0.02	0.006 ± 0.001	0.99 ± 0.02
Zn	0.29 ± 0.01	4.4 ± 0.2	0.16 ± 0.01	2.4 ± 0.1

Table S6. Metal analysis of YPD/Tris medium treated with 125 μg/mL of S100A12 in the absence and presence of a 2-mM Ca(II) supplement (mean ± SEM, *n* = 4).

**Element**	**–Ca(II), ppm**	**–Ca(II), μM**	**+Ca(II), ppm**	**+Ca(II), μM**
Mg	3.0 ± 0.2	120 ± 8	3.1 ± 0.2	130 ± 10
Ca	3.13 ± 0.06	78 ± 2	85 ± 16	2100 ± 400
Mn	0.008 ± 0.001	0.15 ± 0.01	0.013 ± 0.002	0.23 ± 0.04
Fe	0.129 ± 0.006	2.3 ± 0.1	0.13 ± 0.04	2.3 ± 0.7
Co	0.007 ± 0.001	0.110 ± 0.007	0.007 ± 0.001	0.12 ± 0.02
Ni	0.009 ± 0.001	0.14 ± 0.02	0.014 ± 0.004	0.23 ± 0.07
Cu	0.011 ± 0.003	0.17 ± 0.06	0.009 ± 0.002	0.14 ± 0.03
Zn	0.15 ± 0.09	2.4 ± 0.5	0.040 ± 0.1	0.62 ± 2

Table S7. Metal analysis of YPD/Tris medium treated with 250 μg/mL of S100A12 in the presence and absence of a 2-mM Ca(II) supplement (mean ± SEM, n = 4).

**Element**	**–Ca(II), ppm**	**–Ca(II), μM**	**+Ca(II), ppm**	**+Ca(II), μM**
Mg	3.6 ± 0.4	150 ± 21	4.1 ± 0.8	170 ± 33
Ca	3.9 ± 0.6	100 ± 17	120 ± 11	2900 ± 300
Mn	0.009 ± 0.001	0.17 ± 0.02	0.008 ± 0.003	0.14 ± 0.05
Fe	0.16 ± 0.02	2.9 ± 0.4	0.16 ± 0.02	2.9 ± 0.3
Co	0.008 ± 0.001	0.14 ± 0.02	0.008 ± 0.001	0.14 ± 0.02
Ni	0.012 ± 0.002	0.20 ± 0.04	0.013 ± 0.005	0.21 ± 0.08
Cu	0.010 ± 0.002	0.15 ± 0.03	0.009 ± 0.003	0.12 ± 0.03
Zn	0.07 ± 0.03	1.1 ± 0.5	0.05 ± 0.01	0.7 ± 0.2

Table S8. Metal analysis of YPD/Tris medium treated with 250 μg/mL of S100A12 ΔHis$_3$Asp in the absence and presence of a 2-mM Ca(II) supplement (mean ± SEM, n = 4).

**Element**	**–Ca(II), ppm**	**–Ca(II), μM**	**+Ca(II), ppm**	**+Ca(II), μM**
Mg	3.62 ± 0.06	148 ± 2	3.8 ± 0.2	158 ± 7
Ca	4.0 ± 0.1	100 ± 3	121 ± 4	3000 ± 80
Mn	0.007 ± 0.003	0.14 ± 0.05	0.007 ± 0.002	0.13 ± 0.02
Fe	0.14 ± 0.01	2.6 ± 0.2	0.160 ± 0.007	2.8 ± 0.1
Co	0.008 ± 0.000	0.040 ± 0.000	0.009 ± 0.000	0.043 ± 0.002
Ni	0.016 ± 0.000	0.271 ± 0.009	0.020 ± 0.000	0.313 ± 0.005
Cu	0.008 ± 0.000	0.13 ± 0.01	0.006 ± 0.000	0.09 ± 0.01
Zn	0.48 ± 0.06	7.3 ± 0.6	0.46 ± 0.06	7.2 ± 0.6

Table S9. Metal analysis of untreated TSB/Tris medium in the absence and presence of a 2-mM Ca(II) supplement (mean ± SEM, n = 4).

**Element**	**–Ca(II), ppm**	**–Ca(II), μM**	**+Ca(II), ppm**	**+Ca(II), μM**
Mg	3.5 ± 0.2	140 ± 6	3.7 ± 0.2	150 ± 10
Ca	3.5 ± 0.1	90 ± 2	90 ± 12	2300 ± 300
Mn	0.01 ± 0.00	0.14 ± 0.03	0.006 ± 0.002	0.150 ± 0.009
Fe	0.16 ± 0.02	2.9 ± 0.3	0.17 ± 0.01	3.0 ± 0.2
Co	0.022 ± 0.001	0.037 ± 0.002	0.002 ± 0.000	0.04 ± 0.01
Ni	0.02 ± 0.00	0.28 ± 0.07	0.001 ± 0.000	0.211 ± 0.009
Cu	0.01 ± 0.00	0.14 ± 0.04	0.009 ±0.002	0.12 ± 0.04
Zn	0.35 ± 0.02	5.3 ± 0.2	0.34 ± 0.01	5.1 ± 0.2

Table S10. Metal analysis of TSB/Tris medium treated with 62.5 μg/mL of S100A12 in the absence and presence of a 2-mM Ca(II) supplement (mean ± SEM, *n* = 4).

Element	–Ca(II), ppm	–Ca(II), μM	+Ca(II), ppm	+Ca(II), μM
Mg	3.3 ± 0.2	140 ± 9	3.4 ± 0.3	140 ± 13
Ca	3.5 ± 0.2	90 ± 5	97 ± 7	2400 ± 200
Mn	0.009 ± 0.002	0.17 ± 0.05	0.008 ± 0.001	0.15 ± 0.02
Fe	0.14 ± 0.02	2.5 ± 0.4	0.14 ± 0.03	2.4 ± 0.5
Co	0.002 ± 0.000	0.037 ± 0.003	0.002 ± 0.000	0.033 ± 0.002
Ni	0.023 ± 0.002	0.39 ± 0.03	0.015 ± 0.001	0.26 ± 0.02
Cu	0.008 ± 0.002	0.13 ± 0.03	0.008 ± 0.002	0.13 ± 0.02
Zn	0.38 ± 0.02	5.8 ± 0.3	0.09 ± 0.02	1.4 ± 0.4

Table S11. Metal analysis of TSB/Tris medium treated with 125 μg/mL of S100A12 in the absence and presence of a 2-mM Ca(II) supplement (mean ± SEM, *n* = 4).

Element	–Ca(II), ppm	–Ca(II), μM	+Ca(II), ppm	+Ca(II), μM
Mg	3.8 ± 0.3	160 ± 13	3.9 ± 0.3	160 ± 14
Ca	3.2 ± 0.2	80 ± 6	110 ± 20	2700 ± 400
Mn	0.007 ± 0.002	0.13 ± 0.04	0.007 ± 0.001	0.13 ± 0.02
Fe	0.16 ± 0.01	2.8 ± 0.2	0.14 ± 0.02	2.5 ± 0.03
Co	0.022 ± 0.000	0.040 ± 0.002	0.003 ± 0.000	0.047 ± 0.008
Ni	0.025 ± 0.009	0.4 ± 0.1	0.014 ± 0.001	0.24 ± 0.02
Cu	0.008 ± 0.001	0.13 ± 0.02	0.009 ± 0.002	0.15 ± 0.03
Zn	0.227 ± 0.02	3.5 ± 0.3	0.190 ± 0.004	0.29 ± 0.06

Table S12. Metal analysis of TSB/Tris medium treated with 250 μg/mL of S100A12 in the absence and presence of a 2-mM Ca(II) supplement (mean ± SEM, *n* = 4).

	ppm	μM	ppm	μM
Mg	3.5 ± 0.3	150 ± 10	3.7 ± 0.4	150 ± 20
Ca	2.9 ± 0.3	73 ± 8	110 ± 10	2800 ± 300
Mn	0.007 ± 0.001	0.13 ± 0.02	0.007 ± 0.002	0.13 ± 0.03
Fe	0.12 ± 0.02	2.2 ± 0.4	0.15 ± 0.2	2.7 ± 0.3
Co	0.002 ± 0.000	0.027 ± 0.002	0.002 ± 0.000	0.035 ± 0.003
Ni	0.015 0.002	0.26 ± 0.03	0.013 ± 0.001	0.224 ± 0.008
Cu	0.008 ± 0.002	0.12 ± 0.02	0.007 ± 0.001	0.10 ± 0.01
Zn	0.06 ± 0.04	0.9 ± 0.6	0.019 ± 0.003	0.29 ± 0.04

Table S13. Metal analysis of TSB/Tris medium treated with 250 μg/mL of S100A12 ΔHis$_3$Asp in the absence and presence of a 2-mM Ca(II) supplement (mean ± SEM, *n* = 4).

Element	–Ca(II), ppm	–Ca(II), μM	+Ca(II), ppm	+Ca(II), μM
Mg	3.62 ± 0.06	148 ± 2	3.8 ± 0.2	158 ± 7
Ca	4.0 ± 0.1	100 ± 3	121 ± 4	3000 ± 80
Mn	0.007 ± 0.003	0.14 ± 0.05	0.007 ± 0.002	0.13 ± 0.02
Fe	0.14 ± 0.01	2.6 ± 0.2	0.160 ± 0.007	2.8 ± 0.1
Co	0.002 ± 0.000	0.040 ± 0.000	0.003 ± 0.000	0.043 ± 0.002
Ni	0.016 ± 0.000	0.271 ± 0.009	0.020 ± 0.000	0.313 ± 0.005
Cu	0.012 ± 0.002	0.20 ± 0.02	0.010 ± 0.000	0.16 ± 0.01
Zn	0.36 ± 0.02	5.4 ± 0.3	0.36 ± 0.02	5.5 ± 0.3

Table S14. Strains and growth conditions employed in this work.

Strain	Source	Culture conditions[a]
Candida albicans SC5314	Linquist Lab (Whitehead)	YPD/Tris (32:68 v/v), 37 °C, 150 rpm
Candida glabrata ATCC 200918	ATCC	YPD/Tris (32:68 v/v), 37 °C, 150 rpm
Candida krusei ATCC 200917	ATCC	YPD/Tris (32:68 v/v), 37 °C, 150 rpm
Candida tropicalis ATCC MYA-3404	ATCC	YPD/Tris ((32:68 v/v), 37 °C, 150 rpm
E. coli K-12	Keio Collection	TSB/Tris (32:68 v/v), 37 °C, 150 rpm
E. coli K-12 ΔznuA	Keio Collection	TBS/Tris (32:68 v/v), 37 °C, 150 rpm
Lactobacillus plantarum WCSF1	ATCC	MRS/Tris (32:68 v/v). 30 °C, 150 rpm
Listeria monocytogenes ATCC 19115	ATCC	BHI/Tris (32:68 v/v), 30 °C, 150 rpm
P. aeruginosa PAO1	Manoil Laboratory[b]	TBS/Tris (32:68 v/v), 37 °C, 150 rpm
P. aeruginosa PAO1 ΔznuA	Manoil Laboratory[b]	TBS/Tris (32:68 v/v), 37 °C, 150 rpm
S. aureus ATCC 25923	ATCC	Tris : TSB-dextrose, 37 °C, 150 rpm

[a] TSB-dextrose and BHI were obtained from Becton Dickinson (BD). YPD was prepared in-house. All medium contained dextrose. These are the culture conditions employed for the antimicrobial activity assays. [b] University of Washington (Seattle, WA).

Figures S1-S3 detail standard protein characterization.

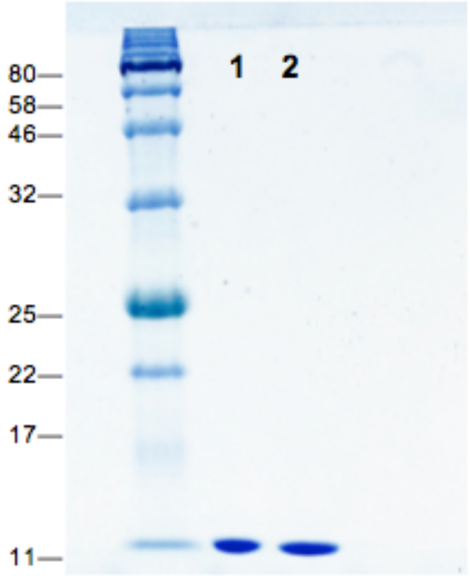

Figure S1. SDS-PAGE (15% acrylamide Tris-HCl, glycine gel) visualized with Coomassie Blue of purified S100A12 (lane 1) and S100A12 ΔHis_3Asp (lane 2) used in this study. The ladder is P7712S from New England BioLabs. The S100A12 monomer is 10.5 kDa.

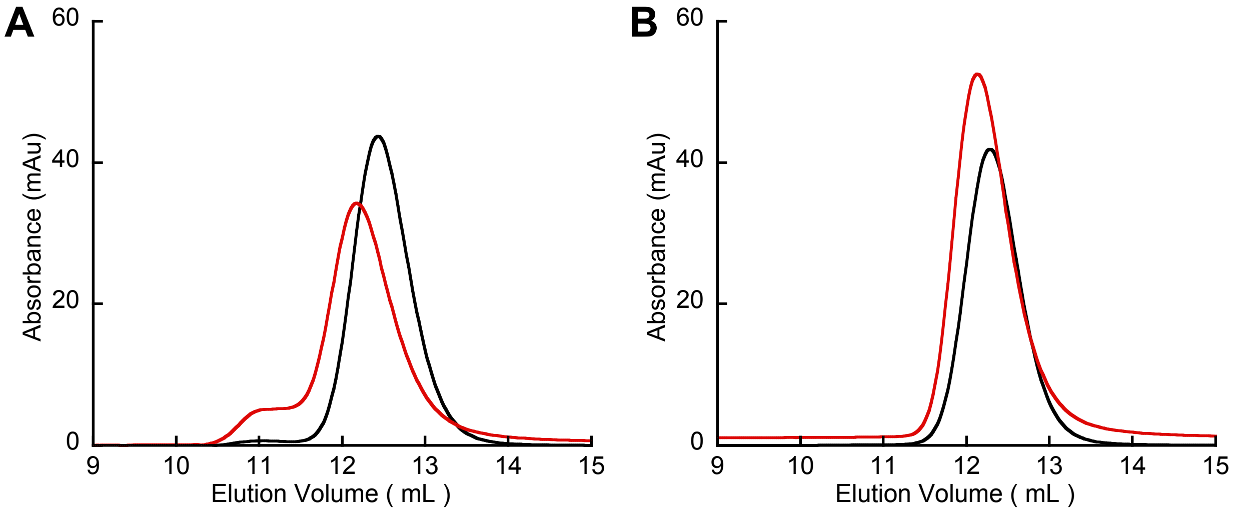

Figure S2. Analytical SEC chromatograms of 30 μM S100A12 (A) and 30 μM S100A12 ΔHis_3Asp (B) in the absence (black) and presence (red) of 2 mM Ca(II) in the sample and running buffer (75 mM HEPES, 100mM NaCl, pH 7.0). Table S3 lists the elution volumes and calculated molecular weights.

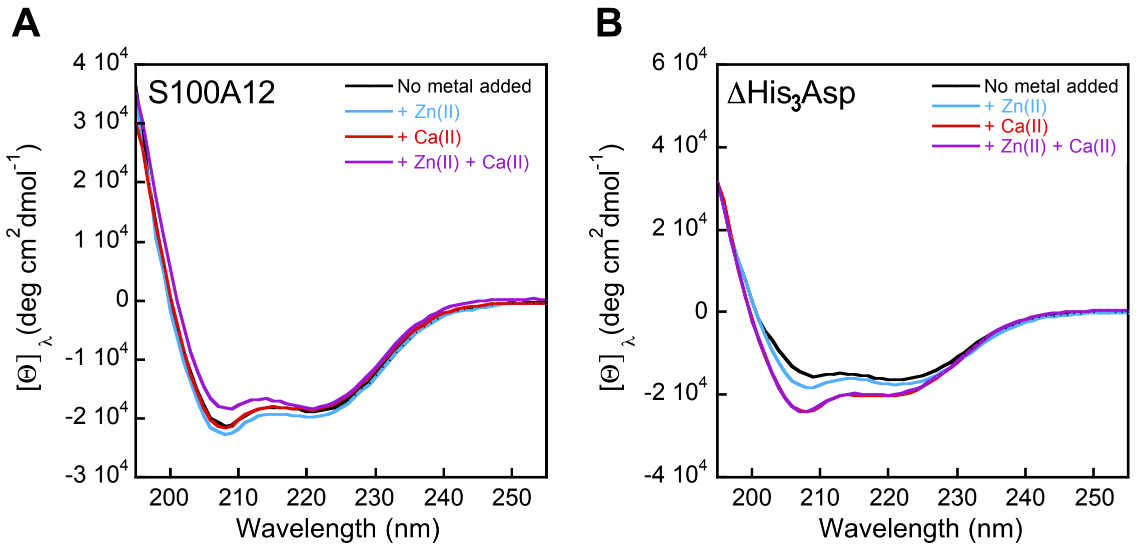

Figure S3. Circular dichroism spectra of 10 μM S100A12 (A) and S100A12 ΔHis_3Asp (B) in the absence and presence of divalent cations (1 mM Tris-HCl, pH 7.0, T = 25 °C). Black trace, without metal addition; blue trace, in the presence of 20 μM Zn(II); red trace, in the presence of 2 mM Ca(II); purple trace, in the presence of 20 μM Zn(II) and 2 mM Ca(II).

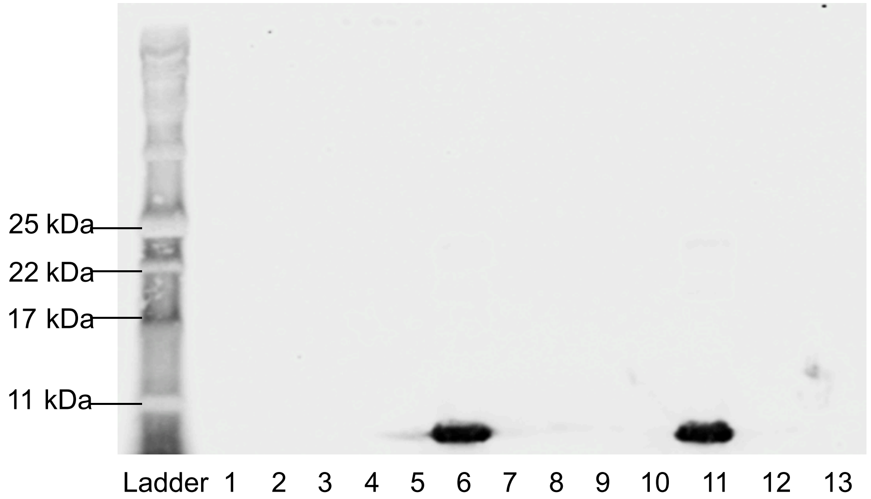

Figure S4. Western blot of the retentate and flow through obtained from S100A12-treated YPD/Tris medium. Medium without S100A12 treatment retentate (lane 2), medium without S100A12 treatment flow through (lane 4), Medium treated with 250 μg/mL S100A12 -Ca(II) retentate (lane 6), Medium treated with 250 μg/mL S100A12 -Ca(II) flow through (lane 8), medium treated with 250 μg/mL S100A12 +Ca(II) retentate, (lane 11), medium treated with 250 μg/mL S100A12 +Ca(II) flow through (lane 13). No sample was loaded in the other lanes (lanes 1, 3, 5, 7, 9, 10, and 12). The ladder is P7712S from New England BioLabs.

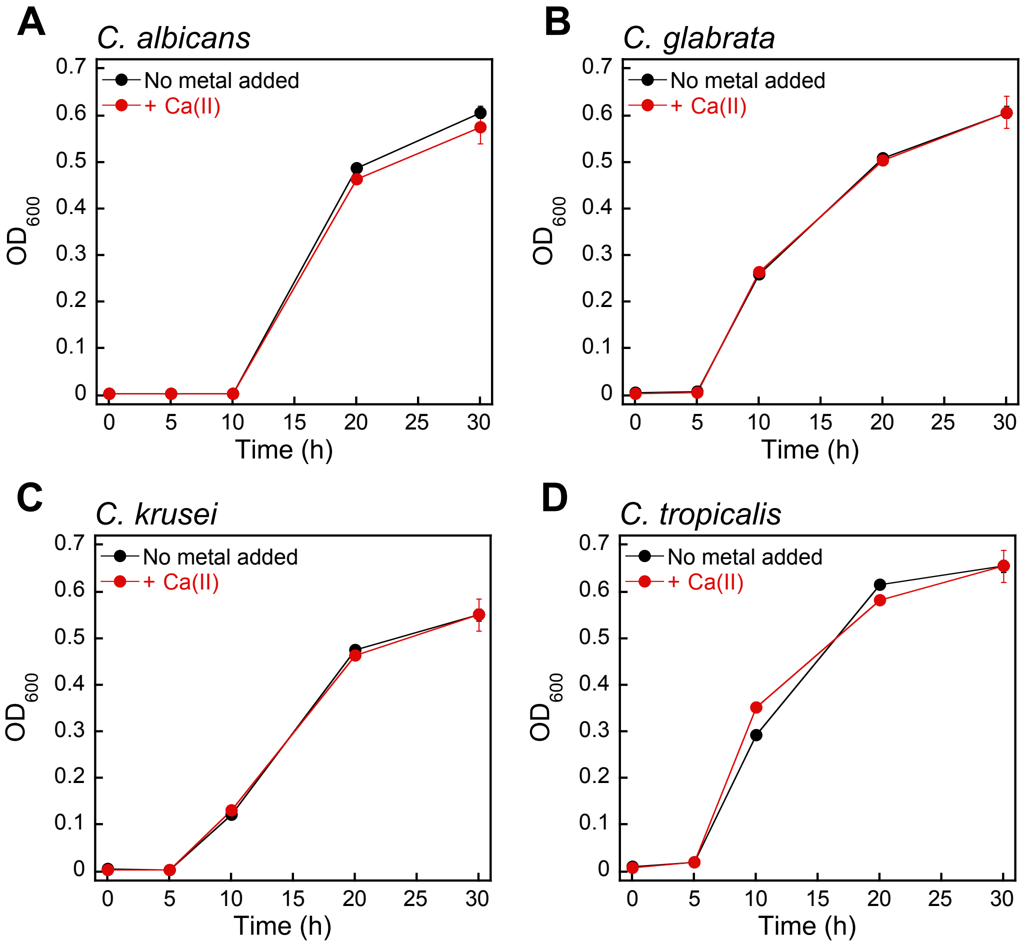

Figure S5. Growth curves of *C albicans* SC 5314 (A), *C. glabrata* ATCC 200918 (B), *C. krusei* ATCC 200917 (C) and *C.* ATCC MYA-3404 (D) in the absence (black trace) and presence (red trace) of a 2-mM Ca(II) supplement (mean ± SEM, *n* = 3).

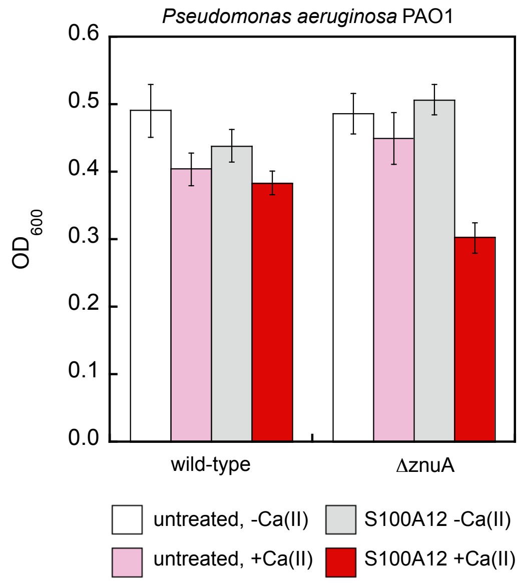

Figure S6. *P. aeruginosa* PAO1 antimicrobial activity data for S100A12 in the absence (black) and presence (red) of a 2-mM Ca(II) supplement, t = 20 h (mean ± SEM, *n* = 3).

The *znuA* gene is not annotated in the Seattle *Pseudomonas aeruginosa* PAO1 Mutant Library. The *znuA* gene was found by searching "znuA" at pseudomonas.com. Gene with locus tag PA5498 is annotated with the name "znuA." BLAST analysis revealed that this gene is a homologue to other *znuA* genes. The Seattle *Pseudomonas aeruginosa* PAO1 Transposon Mutant Library was searched and five strains with a *znuA* mutation were found. One of these strains (mutant: phoAbp01q2A01, gene: *PA5498*) was employed in this study. The sequence of the ZnuA protein deduced from gene *PA5498* for *P. aeruginosa* PAO1 was blasted against two proteins identified as ZnuA transporters (accession numbers EOT09947 and EOT14059) in *P. aeruginosa* PA14. ZnuA PAO1 was found to share 100% and 72% sequence homology with the ZnuA proteins EOT09947 and EOT14059, respectively. A recent report also identified *PA5498* as ZnuA.[10]

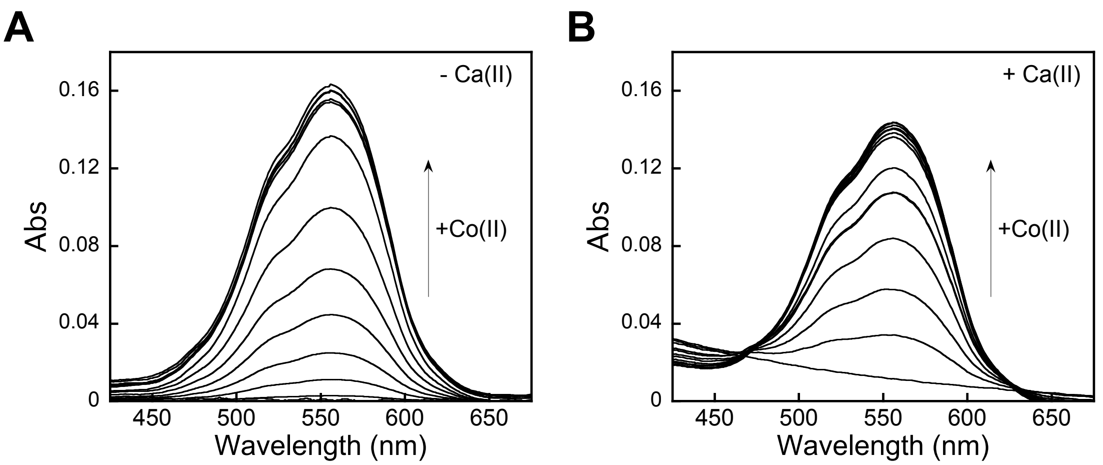

Figure S7. Optical absorption spectra of S100A12 (200 μM) titrated with 0−4 equiv. of Co(II) at pH 7.0 (75 mM HEPES, 100 mM NaCl) and 25 °C, in the absence (A) and presence (B) of 40 equiv. of Ca(II).

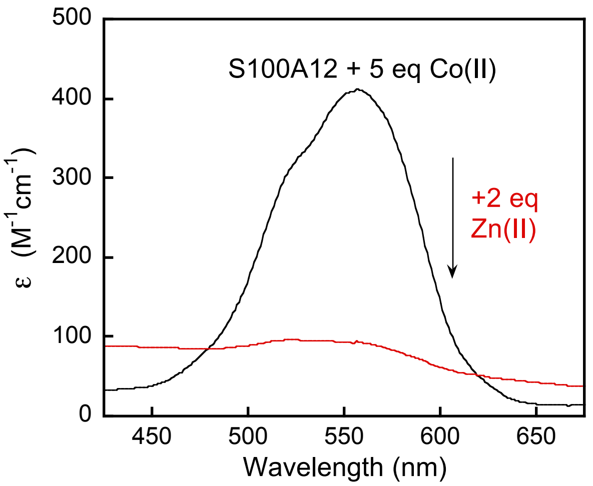

Figure S8. Optical absorption spectra showing displacement of Co(II) from 200 μM S100A12 by Zn(II) addition at pH 7.0 (75 mM HEPES, 100 mM NaCl) and T = 25 °C. The increase in baseline with Zn(II) addition indicates the formation of some precipitate.

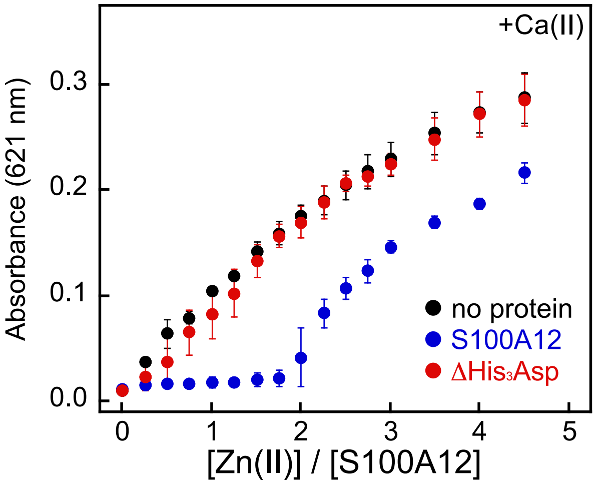

Figure S9. Response of Zincon (20 μM) to Zn(II) in the presence of S100A12 or ΔHis$_3$Asp (10 μM) and 400 μM Ca(II) at pH 7.0 (75 mM HEPES, 100 mM NaCl) and 25 °C (mean ±SDM, *n*=3). The Zn(II)-Zincon complex exhibits an absorbance maximum at 621 nm.

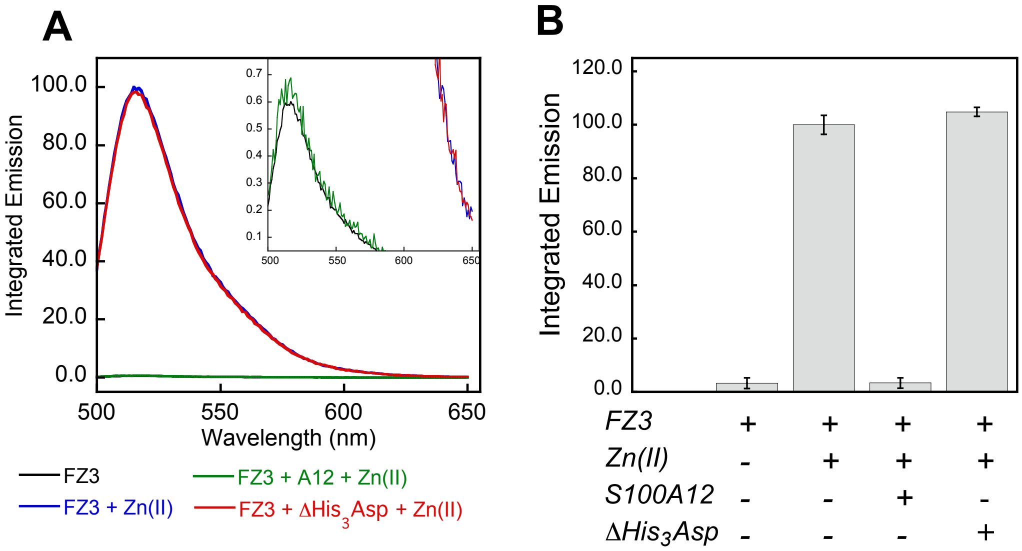

Figure S10. S100A12 outcompetes FZ3 for Zn(II). Fluorescence response of 2 μM FZ3 to 2 μM Zn(II) in the presence of 2 μM S100A12 or S100A12 ΔHis$_3$Asp at pH 7.0 (75 mM HEPES, 100 mM NaCl) and 25 °C. (A) An ≈36-fold fluorescent enhancement is observed for FZ3 in the absence of protein (blue trace) and in the presence of ΔHis$_3$Asp (red trace) upon addition of 1 equiv. of Zn(II). Negligible fluorescent response is observed for FZ3 in the presence of S100A12 (green trace) upon addition of 1 equiv. of Zn(II) (Inset: Expansion of the y-axis). (B) integrated emission values for the data presented in panel A and replicates. The maximum emission for FZ3 in the presence of 1 equiv. of Zn(II) was normalized to an integrated emission value of 100, and the remaining emission spectra were scaled accordingly (mean ± SEM, *n*=3).

Supplementary References

1. M. B. Brophy, T. G. Nakashige, A. Gaillard and E. M. Nolan, *J. Am. Chem. Soc.*, 2013, **135**, 17804-17817.
2. F. Citiulo, I. D. Jacobsen, P. Miramón, L. Schild, S. Brunke, P. Zipfel, M. Brock, B. Hube and D. Wilson, *PLoS Pathog.*, 2012, **8**, e1002777.
3. M. B. Brophy, J. A. Hayden and E. M. Nolan, *J. Am. Chem. Soc.*, 2012, **134**, 18089-18100.
4. T. G. Nakashige, B. Zhang, C. Krebs and E. M. Nolan, *Nat. Chem. Biol.*, 2015, **11**, 765-771.
5. W. Maret and B. L. Vallee, *Proc. Natl. Acad. Sci. U. S. A.*, 1998, **95**, 3478-3482.
6. C. Talmard, A. Bouzan and P. Faller, *Biochemistry*, 2007, **46**, 13658-13666.
7. T. J. B. Simons, *J. Biochem. Biophys. Methods*, 1993, **27**, 25-37.
8. M. V. Golynskiy, W. A. Gunderson, M. P. Hendrich and S. M. Cohen, *Biochemistry*, 2006, **45**, 15359-15372.
9. A. Krężel and W. Maret, *J. Am. Chem. Soc.*, 2007, **129**, 10911-10921.
10. V. G. Pederick, B. A. Eijkelkamp, S. L. Begg, M. P. Ween, L. J. McAllister, J. C. Paton and C. A. McDevitt, *Sci. Rep.* 2015, **5**, 13139.
